# Diel Vertical Dynamics of Gelatinous Zooplankton (Cnidaria, Ctenophora and Thaliacea) in a Subtropical Stratified Ecosystem (South Brazilian Bight)

**DOI:** 10.1371/journal.pone.0144161

**Published:** 2015-12-04

**Authors:** Miodeli Nogueira Júnior, Frederico Pereira Brandini, Juan Carlos Ugaz Codina

**Affiliations:** 1 Departamento de Sistemática e Ecologia, Universidade Federal da Paraíba, João Pessoa, Paraíba, Brasil; 2 Departamento de Oceanografia Biológica, Instituto Oceanográfico, Universidade de São Paulo, São Paulo, Brasil; 3 Programa de Pós Graduação em Zoologia, Departamento de Zoologia, Universidade Federal do Paraná, Curitiba, Paraná, Brasil; The Evergreen State College, UNITED STATES

## Abstract

The diel vertical dynamics of gelatinous zooplankton in physically stratified conditions over the 100-m isobath (~110 km offshore) in the South Brazilian Bight (26°45’S; 47°33’W) and the relationship to hydrography and food availability were analyzed by sampling every six hours over two consecutive days. Zooplankton samples were taken in three depth strata, following the vertical structure of the water column, with cold waters between 17 and 13.1°C, influenced by the South Atlantic Central Water (SACW) in the lower layer (>70 m); warm (>20°C) Tropical Water in the upper 40 m; and an intermediate thermocline with a deep chlorophyll-*a* maximum layer (0.3–0.6 mg m^-3^). Two distinct general patterns were observed, emphasizing the role of (i) physical and (ii) biological processes: (i) a strong influence of the vertical stratification, with most zooplankton absent or little abundant in the lower layer. The influence of the cold SACW on the bottom layer apparently restricted the vertical occupation of most species, which typically inhabit epipelagic warm waters. Even among migratory species, only a few (*Aglaura hemistoma*, *Abylopsis tetragona* eudoxids, *Beroe* sp., *Thalia democratica*, *Salpa fusiformis*) crossed the thermocline and reached the bottom layer. (ii) A general tendency of partial migrations, with variable intensity depending on the different species and developmental stages; populations tended to be more widely distributed through the water column during daylight, and to become more aggregated in the upper layer during the night, which can be explained based on the idea of the “hunger-satiation hypothesis”, maximizing feeding and minimizing the chances of being predated.

## Introduction

The vertical distribution of plankton assemblages and their variations in space and time are important for understanding the organization and dynamics of pelagic communities, and the vertical flux of organic matter through the water column. Planktonic organisms may change their vertical position according to their sex, stage of development, season, and/or period of the day; this last is the most common behavior [1–6]. Diel vertical migration (DVM) is widespread among zooplankton and may be the largest animal migration on the planet [4]. Although other patterns may occur, zooplankters most typically ascend early in the evening and descend at dawn [1–6].

Several biological and physical factors may influence these migrations, such as the abundance and distribution of predators and food, illumination levels, hydrographic structure, and size and nutritional condition of individuals [1, 4–10], among others. However, our knowledge of marine zooplankton ecology in general and of DVM in particular is generally derived from studies of crustaceans and in temperate environments, with an increasing need to focus on tropical [5, 11] and subtropical situations, as well as on other groups such as gelatinous forms [12, 13]. These organisms are common components of coastal, shelf and oceanic pelagic ecosystems, and recurrently play significant roles as primary or secondary consumers due to their high biomass and feeding rates [14, 15]. In addition, thaliaceans produce large, rapidly sinking fecal pellets with high organic content, which are important in exporting pelagic organic matter to deep waters and/or benthos [15]. Therefore, detailed understanding of their quantitative distribution patterns, including the vertical component, is important to recognize ecological processes and energy flow in the water column.

Since the pioneering study of Russell [16], the diel vertical distribution of several taxonomic groups of gelatinous zooplankters has been described (e.g. [2, 7, 8, 17–20]). These studies mostly targeted particular species and/or groups, hampering recognition of general assemblage patterns that may help to recognize the main environmental drivers. On the Brazilian continental shelf only two old studies have addressed the issue [2, 21]. However, they studied only a specific group (hydromedusae [2] or salps [21]), and their samples were taken either over the shallow shelf [2] where water-column stratification is usually absent or weak, or diurnal and nocturnal samples were not taken at the same station or even on the same day [21] hindering accurate interpretation of DVM patterns.

The shelf of the South Brazilian Bight is a good model for investigations of DVM of gelatinous organisms. The diversity of gelatinous zooplankton is high [22], representative of a tropical situation on the mid- to outer shelf, where the water column is typically a well-defined three-layered system year-round; i) the upper mixed layer is mostly influenced by the warm (>20°C) oligotrophic Tropical Water, occasionally mixed with the less-saline Coastal Water depending on the wind regime; ii) the bottom layer is influenced by the cold (<17°C) and nutrient-rich South Atlantic Central Water (SACW); iii) the intermediate layer is the boundary between these environments, with the presence of a thermocline and a deep chlorophyll-*a* maximum layer [23–25]. Within this three-layered hydrographic structure, we analyzed the diel vertical dynamics of the assemblage of gelatinous zooplankton (Cnidaria, Ctenophora and Thaliacea) over two consecutive days, in order to test for diurnal and nocturnal differences in their vertical distributions, and to explore possible relationships to oceanographic characteristics and food availability.

## Materials and Methods

### Field work

**Fig 1:**
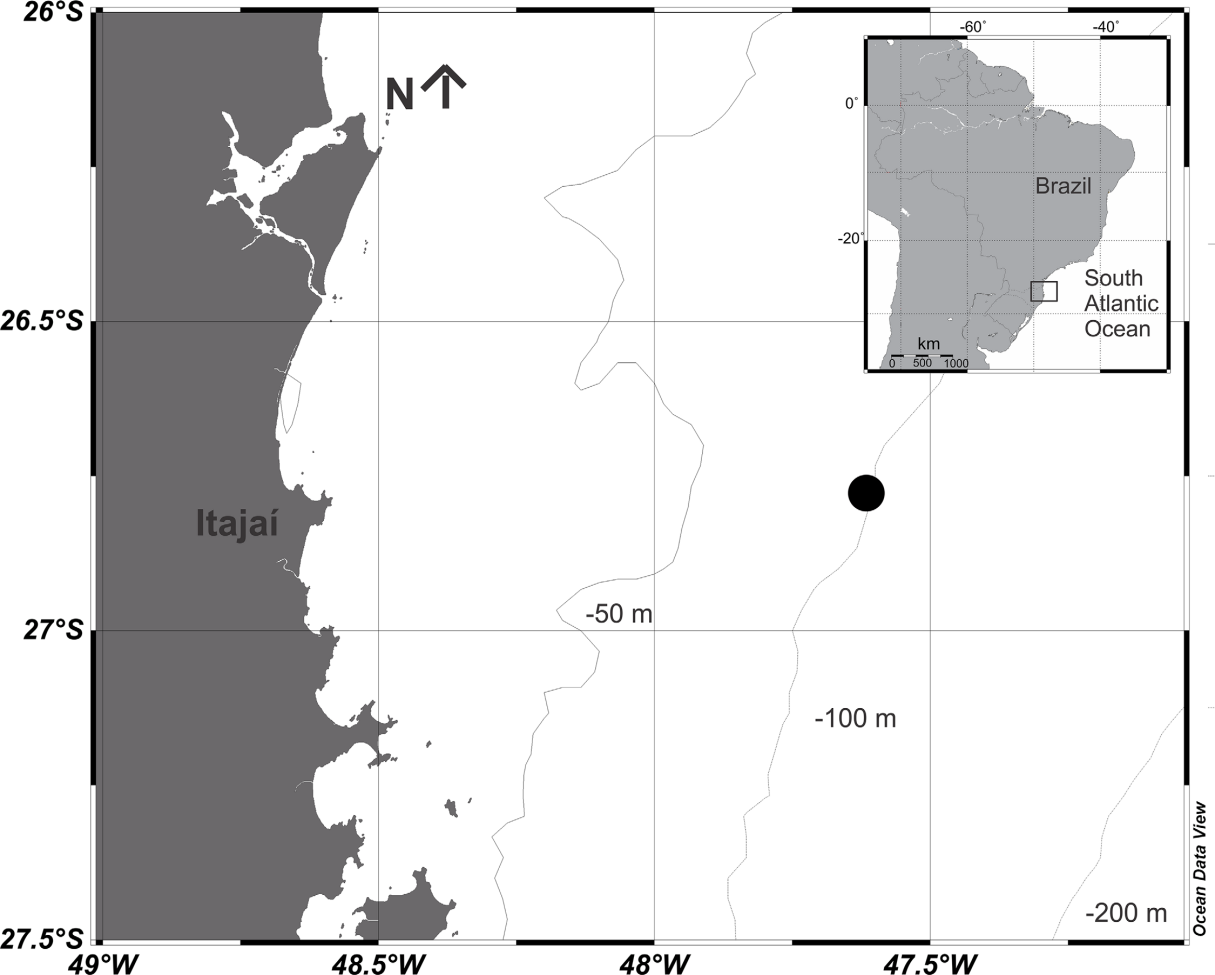
Map of the study site showing the station sampled. Generated using Ocean Data View software (after Schlitzer [98]).

The data were obtained with the support of the R.V. “Soloncy Moura” (Brazilian Ministry of the Environment) at a fixed station over the 100-m isobath (~110 km offshore) in the southern part of the South Brazilian Bight, off Itajaí Harbor, Santa Catarina State (26°45’S; 47°33’W; Fig 1). Sampling began on October 16 2007 at 06:00 and ended on October 18 at 01:15. Temperature and salinity vertical profiles were regularly obtained with the InterOcean CTD/S4 multiparameter probe throughout the sampling period. Sigma-t was calculated from the temperature and salinity data. The stratification index, defined as the difference between the sigma-t of the bottom and that of the subsurface (-5 m), was also calculated, and was classified as weak (<0.5), moderate (0.5–2) or strong (>2). Chlorophyll-*a* concentrations and light intensity (as photosynthetically active radiation, PAR) were regularly obtained with the Profiling Natural Fluorometer model PNF-300 (Biospherical Instruments).

Zooplankton was sampled with vertical hauls, using a WP2 net with 0.5-m mouth diameter and 200-μm mesh size, equipped with a calibrated flowmeter and closing mechanism. Hauls were performed approximately every six hours (early morning: ~06:00–08:00; noon: ~12:00–14:00; early night: ~18:00–20:00; midnight: ~24:00–02:00) in triplicate, in each of three strata defined according to the vertical hydrographic structure, totaling 72 samples: (i) in the warmer upper mixed layer (UML; <40 m), defined by temperatures >20°C, which is the lower limit of the Tropical Water that mostly characterizes the UML on the outer shelf off the South Brazilian Bight [23–25]; (ii) at the level of the deep chlorophyll-*a* maximum layer (DCM, between 40–70 m) and encompassing the thermocline, defined by the gradual decrease of temperature and peaks of chlorophyll-*a* concentrations; and (iii) in the colder bottom layer under SACW dominance (BL; 70 m–near bottom), defined by temperatures <17°C [23–25].

Samples were visually inspected soon after retrieval of the nets for large gelatinous organisms (>10 mm), which were separated and identified on board, and subsequently fixed in formalin diluted to 4% with filtered (<30 μm) local sea water. We declare that the field sampling did not involve endangered or protected species and that no specific permission is required by the Brazilian Government.

### Laboratory work

Samples were analyzed in their entirety under a stereomicroscope, and the cnidarians, ctenophores and thaliaceans were identified and quantified. Identification mainly followed the appropriate chapters in the compendium edited by Boltovskoy [26]. For calycophore siphonophores, the number of polygastrics (as anterior nectophores) and eudoxids (as bracts) was counted. For physonects, the number of colonies was estimated by the number of pneumatophores, and when these were absent, nectophores were counted, and the total was divided by ten to roughly approximate the actual number of colonies sampled [27–28]. The abundances were standardized as the number of individuals (or colonies in the case of siphonophores and pyrosomes) per 10 m^-3^ of filtered sea water.

Other zooplankton groups, interpreted as potential food for the gelatinous carnivorous, were quantified from whole samples (chaetognaths and larvaceans) or aliquots (all other taxa) of the first series of triplicate hauls (n = 24 samples). Aliquots (1/4 to 1/32) were taken by successively dividing the samples in half to obtain a minimum of 300 individuals [29].

### Data analysis

The mean vertical position (weighted mean depth) of a given species or developmental stage was estimated using the following equation [5]:

$$\text{weighted mean depth}\;=\Sigma \;\left({\text{d}}_{\text{i}}*{\text{p}}_{\text{i}}\right)\;/\;\Sigma \;\text{d}$$

where p_i_ is the mean depth of the stratum sampled (i), d_i_ is the mean density of the stratum, and d is the total density in the water column. The weighted mean depth is best suited for strata with the same depth range, because wider strata weigh more and bias the index. Notwithstanding, weighted mean depth is widely employed in the literature independently of the homogeneity of strata height (e.g.[18, 20, 30–32]) and is a good index for comparative purposes, particularly where the depths of the strata sampled are constant, as was the case here. A t test was utilized to test the hypothesis that the weighted mean depth of each species or developmental stage changes significantly (p<0.05) between diurnal and nocturnal periods [33].

In order to recognize possible assemblage changes through the diel cycle, two statistical approaches were applied: i) a permutational multivariate analysis of variance (PERMANOVA) was used to test the null hypothesis that the assemblage structure of gelatinous zooplankton did not change with depth stratum (with three levels), period of the day (two levels; diurnal and nocturnal), sample replica (three levels), and possible interactions among these three factors. Significance (P<0.05) was estimated by the pseudo-F statistic and the Monte Carlo permutation, after 999 runs. In case of significant differences, a pairwise test between different levels of significant factor(s) was performed [34, 35]; ii) a hierarchical cluster analysis (group average linking mode) was performed to identify patterns of similar samples and therefore possible changes (or not) in the vertical distribution of assemblages through the daily cycle [36]. Since the PERMANOVA suggested that the factor replica did not contribute significantly to the variance of the data-set (Table 1), and to simplify graphical visualization, averaged values (n = 24) of each of the three replicates were used in the cluster. Both the PERMANOVA and the cluster were based on a Bray-Curtis similarity matrix constructed on the log (x+1) transformed densities of all species; for siphonophores and thaliaceans the different developmental stages were computed separately.

**Table 1:** Summary of the PERMANOVA. This analysis tests differences in quantitative taxonomic composition of gelatinous zooplankton assemblage considering depth stratum (with three levels), day period (two levels, diurnal and nocturnal), and sample replicate (three levels) as factors. Differences are considered significant if P and MC (P) <0.01 (in bold). df = degrees of freedom; SS = sum of squares; MS = mean squares; P = probability associated with the Pseudo F statistic; MC (P) = probability associated with the Monte Carlo randomization procedure.

Factors	df	SS	MS	Pseudo F	P	MC (P)
**Depth stratum**	**2**	**29914**	**14957**	**7.038**	**0.001**	**0.001**
**Day period**	**1**	**7541**	**7541**	**3.548**	**0.001**	**0.001**
Replicate	2	4527	2264	1.065	0.363	0.345
**Depth x day period**	**2**	**14324**	**7162**	**3.37**	**0.001**	**0.001**
Depth x Replicate	4	6924	1731	0.8145	0.814	0.796
Day period x replicate	2	3429	1714	0.807	0.734	0.718
Depth x day period x replicate	4	6487	1622	0.763	0.882	0.862
Residuals	54	<0.0001	2125			
Total	71	<0.0001				

The species richness, total abundance and abundance of dominant taxa/developmental stages of gelatinous zooplankton from each stratum were compared using analysis of variance (ANOVA). In case of significant differences (p<0.05), pairwise comparisons using the t test were performed after applying the Bonferroni correction [33]. These analyses were done for nocturnal and diurnal periods separately.

In order to relate species distribution to possible explanatory variables, we used a constrained ordination analysis [37]. The length of the gradient of the detrended correspondence analysis was small (<3) and therefore we used the Redundancy Analysis following the recommendations of Lepš & Šmilauer [37]. Temperature, salinity, PAR, sigma-t, chlorophyll-*a*, and abundances of the different zooplankton groups were included as possible explanatory variables. Prior to the analysis, the abundance data were transformed by log (x+1), and explanatory variables were centered and standardized [37]. After initial trials, it became clear that the different zooplankton taxa are highly autocorrelated with each other, and therefore only copepods were retained in the model as a measure of food availability for the gelatinous carnivores. Similarly, sigma-t was also excluded because of the high inter-correlation with temperature, both of which defined the physical vertical gradient. These deletions were made in order to avoid inflating the explanatory power of the model due to autocorrelation of the explanatory variables.

## Results

### Oceanographic structure

**Fig 2:**
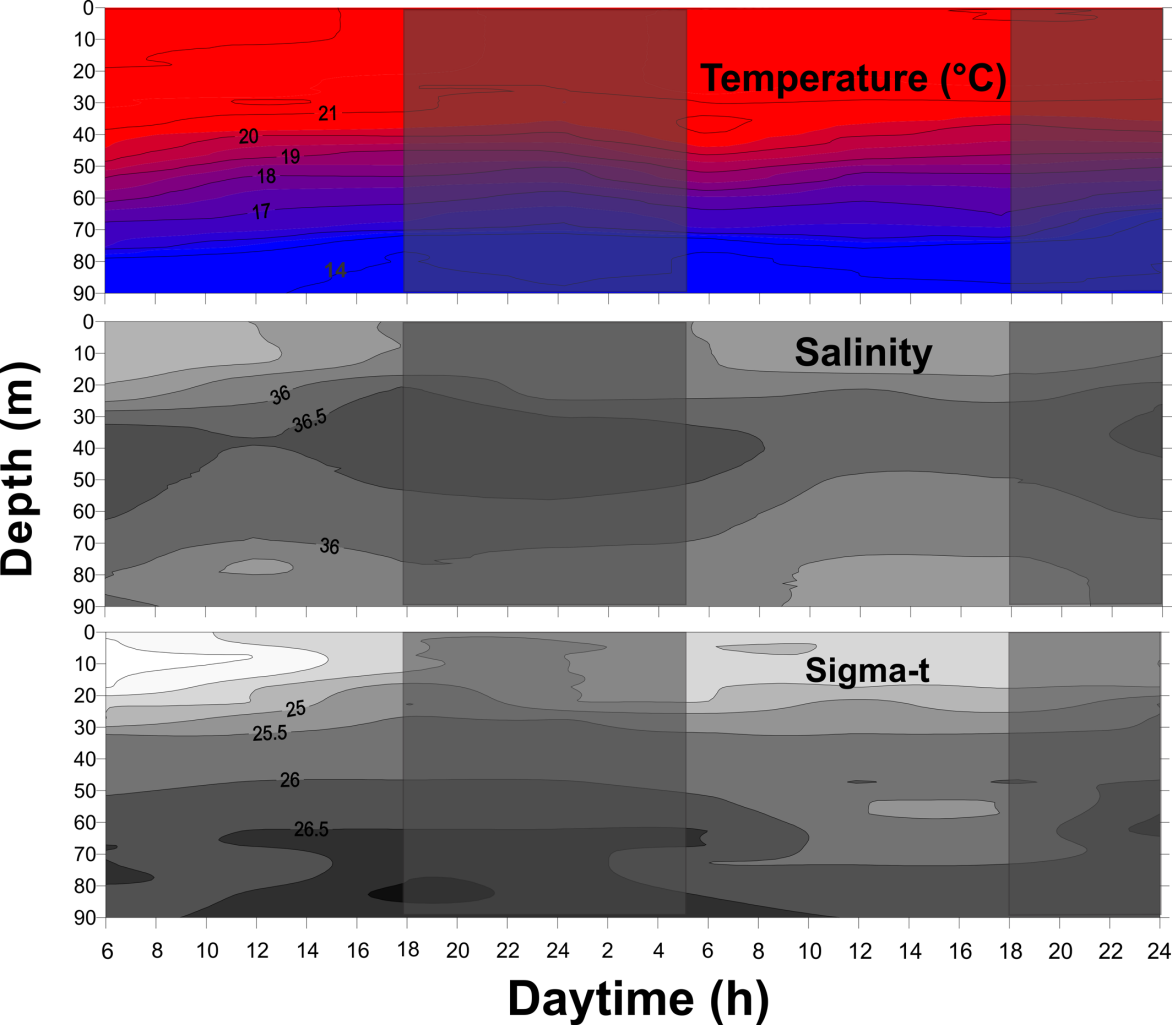
Vertical profiles of physical parameters of the water column. Temperature and salinity data were collected with a CTD/S4 (InterOcean) regularly throughout October 16 and 17, 2007 off South Brazilian Bight. Shadows indicate nocturnal periods. In the temperature panel the isolines are of 1°C, in the salinity and sigma-t panels the isolines are 0.5.

The water column maintained essentially the same vertical hydrographic structure throughout the course of this study. Temperature was high (≥20°C) in the upper 40 m, and decreased downward to <17°C below the 70-m depth, to as low as 13.1°C (Fig 2). A deep salinity maximum layer of >36 was observed between the 30–60 m depths (Fig 2). The sigma-t pattern basically followed that of the temperature. The stratification index ranged from 1.7 at noon of the second day to 2.7 between 06:00 and 20:00 of the first day, averaging (± standard deviation) 2.2±0.4, suggesting a strong physical stratification during most of the sampling period.

**Fig 3:**
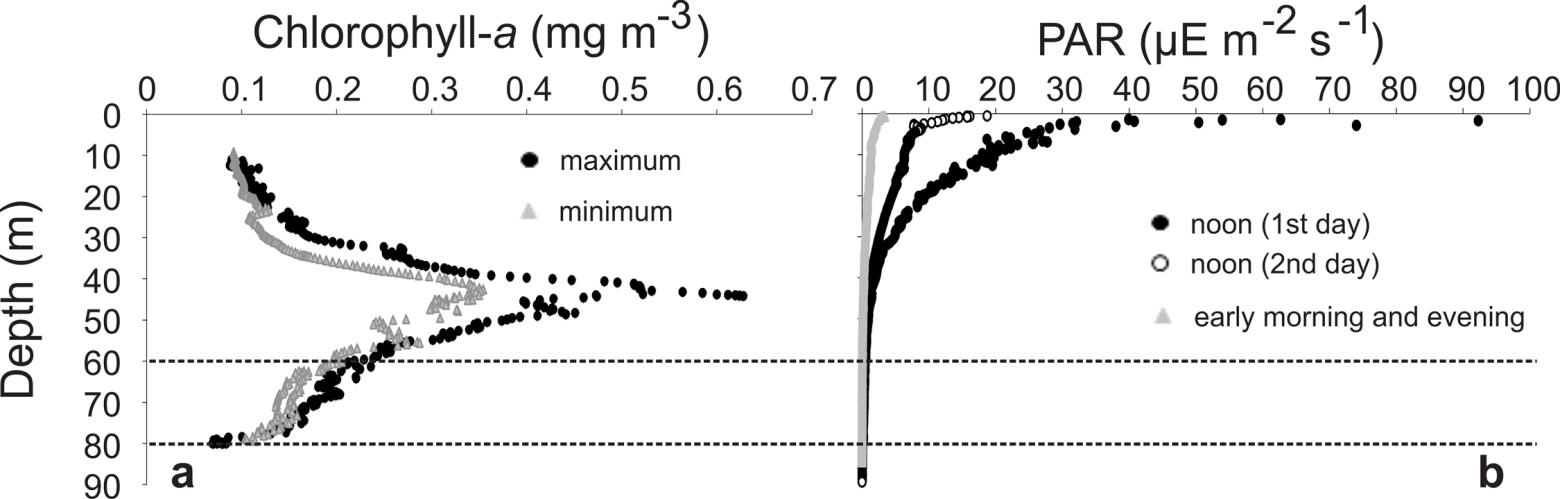
Summary of the vertical profiles of chlorophyll-*a* (a) and light levels (b). Dotted horizontal lines indicate the variation in the euphotic zone (1% of light level). PAR = photosynthetically active radiation.

Chlorophyll-*a* reached maximum concentrations ≥0.3 mg m^-3^ (up to 0.64 mg m^-3^) between 40–60 m depth. Above and below this DCM layer the concentrations were lower, usually <0.15 mg m^-3^ (Fig 3A). Light intensity was higher during the first sampling day, with values exceeding 50 μE.m^-2^.s^-^ in the first 2 m, gradually decreasing to 20 μE.m^-2^.s^-^ in the first 10 m (Fig 3B). The euphotic zone, determined by the 1% surface PAR level, was around 60 to 80 m deep during the two days (Fig 3).

### Gelatinous zooplankton

#### Species composition

A total of 46 species were sampled, 17 medusae, 19 siphonophores, 7 thaliaceans and 3 ctenophores, in addition to unidentifiable larval forms such as actinula, bitentaculata and athorybia, and a few (<1%) damaged unidentified individuals. Thaliaceans were the most abundant gelatinous taxon, comprising 50% of individuals, followed by medusae (25%) and siphonophores (23%). *Doliolum nationalis* comprised 52% of the thaliaceans, while *Aglaura hemistoma* was the most abundant medusa (66%), *Abylopsis tetragona* (33.5%) and *Diphyes bojani* (30%) were the most abundant siphonophores, and small-sized *Beroe* sp. (1 to 9.5 mm high) comprised more than 90% of all ctenophores. The complete list of all species, and their mean diurnal and nocturnal densities and weighted mean depths are shown in S1–S4 Tables.

#### Assemblage structure and vertical dynamics

The PERMANOVA indicated that the structure of the assemblage differed according to depth strata and period of the day, but not according to the different replicates. The interaction among these factors followed a similar pattern, with no significance whenever the factor replicate was included in the analysis (Table 1). The pairwise comparisons suggested that all depth strata differed significantly (P<0.01) from each other, considering either the whole dataset or the diurnal and nocturnal samples separately.

**Fig 4:**
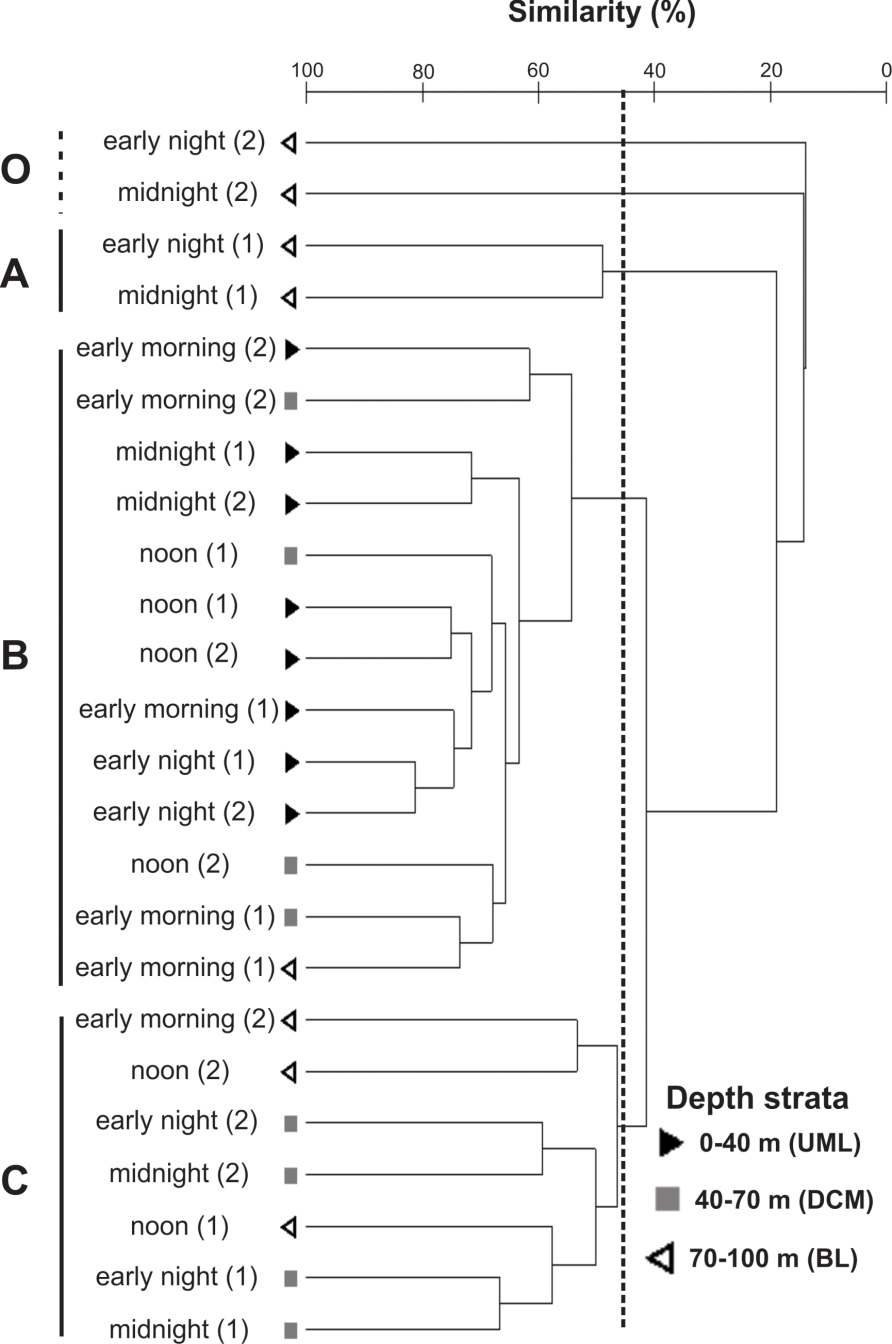
Hierarchical cluster of the samples. Cluster (group average mode) was generated using a Bray-Curtis similarity matrix after abundance data was transformed by log (x+1). Samples represent a mean of three replicate and are labeled according to the period of the day. The symbols indicate the different depth strata sampled according to the legend (UML = upper mixed layer; DCM = deep chlorophyll-*a* maximum layer; BL = bottom layer). The numbers in parenthesis indicate the first (1) or second (2) day sampled. A, B, C are the groups formed in the analysis with >40% of similarity and O = outliers.

Similarly, the general tendencies of sample clustering suggested the existence of vertical differences and temporal changes in the vertical structure of the assemblage (Fig 4): i) the nocturnal BL samples (groups A and O) shared <20% similarity with all other samples; ii) the diurnal DCM samples tended to cluster with the UML samples (both diurnal and nocturnal ones), forming group B; iii) the diurnal BL samples tended to cluster with the nocturnal samples from DCM, forming group C (Fig 4).

**Fig 5:**
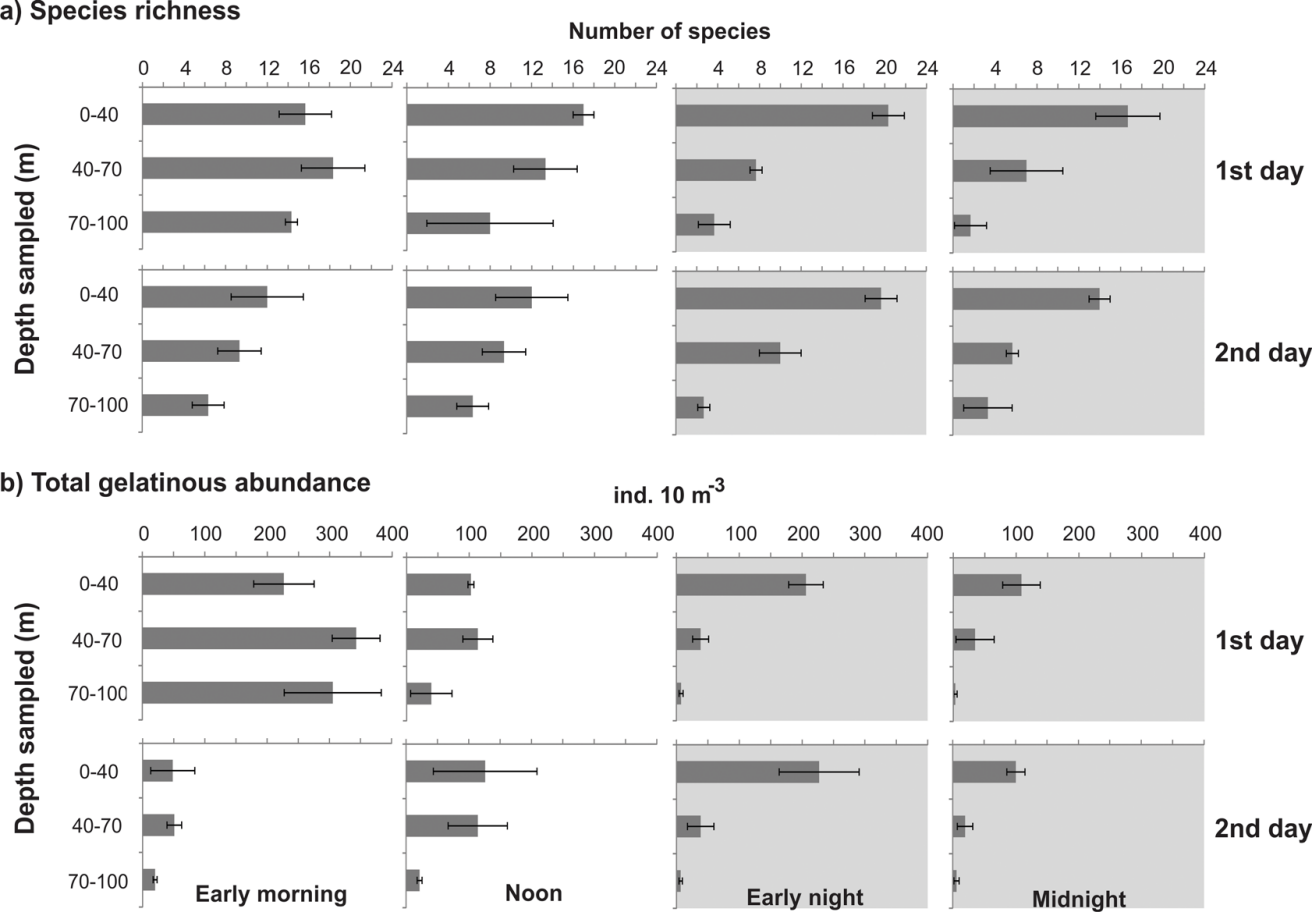
Vertical distribution of gelatinous zooplankton species richness and total abundance. Shadows indicate nocturnal periods. Values are shown as the average ± standard deviation.

Species richness ranged from a mean of <2 in the BL during the first midnight to a mean of ~20 in the UML from the early night samples of both days (Fig 5A). Species richness varied significantly through the water column, and was always higher in the UML and lower in the BL, independently of the period of the day (Table 2; Fig 5A). The differences were more pronounced during the night, when all layers differed significantly from each other (Table 2; p<0.05) and species richness tended to increase in the UML and decrease in the two lower layers compared to diurnal periods. For instance, the mean species richness in the BL ranged from 6 to 14.3 and from 1.7 to 3.7 in the diurnal and nocturnal samples respectively (Fig 5A).

**Table 2:** Summary of gelatinous zooplankton diel vertical distribution on South Brazilian Bight. Species richness and abundance (ind. or col. 10 m^-3^) of total and main gelatinous taxa/developmental stage is shown as average ± standard error (n = 12 on each case). An analysis of variance (ANOVA) was performed to test the effect of depth stratum (UML = upper mixed layer, 0–40 m; DCM = deep chlorophyll maximum layer, 40–70 m; BL = bottom layer, 70–100 m) on each variable considering diurnal and nocturnal data-sets separately. In case of significant differences (in bold; p<0.05) a t test was used to compare each pair of strata after applying the Bonferroni correction, the results indicated by the superscript letter; strata sharing at least one letter do not differ significantly between each other.

	Strata	Diurnal	Nocturnal		Strata	Diurnal	Nocturnal
Species Richness	UML	15.2±1.2^a^	17.7±0.9 ^a^	*A*. *eschscholtzii*	UML	0.9±0.3 ^a^	1.4±0.5 ^a^
	DCM	13.2±1.2 ^a^	7.6±0.7 ^b^	(polygastrics)	DCM	1.0±0.4 ^a^	0.1±0.1 ^b^
	BL	8.7±1.3 ^b^	2.8±0.4 ^c^		BL	0.0 ^b^	0.0 ^b^
	F	**7.843**	**191.9**		F	**3.42**	**7.584**
	p	**0.0016**	**<0.0001**		P	**0.0447**	**0.0019**
Total Abundance	UML	125.9±23.2	160.6±19.5 ^a^	*E*. *hyalinum*	UML	1.2±0.4 ^a^	2.2±0.4 ^a^
	DCM	155.3±34.4	32.8±5.5 ^b^	(eudoxids)	DCM	0.0 ^b^	0.6±0.4 ^b^
	BL	96.5±37.8	5.8±0.9 ^c^		BL	0.3±0.2 ^b^	0.1±0.1 ^b^
	F	0.8228	**54.03**		F	**6.186**	**10.50**
	p	0.4480	**<0.0001**		p	**0.0052**	**0.0003**
*A*. *hemistoma*	UML	6.5±1.2 ^b^	18.4 ±2.4 ^a^	*E*. *hyalinum*	UML	2.2±0.4 ^a^	2.8±0.9 ^a^
	DCM	42.4±12.7 ^a^	3.8±1.2 ^b^	(polygastrics)	DCM	1.1±0.6 ^a^	0.6±0.4 ^b^
	BL	29.6±12.7 ^ab^	0.5±0.2 ^b^		BL	0.1±0.1 ^b^	0.1±0.1 ^b^
	F	**4.14**	**37.84**		F	**6.00**	**6.181**
	p	**0.04**	**<0.0001**		p	**0.0060**	**0.0052**
*C*. *gracilis*	UML	2.6±1.0	5.6±1.9 ^a^	*L*. *subtilis*	UML	0.4±0.2	0.7±0.4
	DCM	1.7±0.5	0.8±0.4 ^b^	(both stages)	DCM	0.7±0.3	0.0
	BL	0.6±0.3	0.0 ^b^		BL	0.8±0.4	0.1±0.1
	F	2.34	**6.943**		F	0.3822	2.919
	p	0.1119	**0.003**		p	0.6853	0.0681
*L*. *tetraphylla*	UML	1.4±0.5	4.0±1.0 ^a^	*M*. *kochii*	UML	1.1±0.3 ^a^	2.2±0.6 ^a^
	DCM	2.7±0.8	1.8±0.7 ^ab^	(both stages)	DCM	1.0±0.3 ^a^	0.1±0.1 ^b^
	BL	1.8±0.9	0.0 ^b^		BL	0.0 ^b^	0.0 ^b^
	F	0.739	**9.656**		F	**4.670**	**12.72**
	p	0.485	**0.0005**		p	**0.0164**	**<0.0001**
*A*. *tetragona*	UML	1.9±0.4 ^b^	15.8±1.9 ^a^	*E*. *spiralis*	UML	1.1±0.3 ^a^	1.2±0.7
(eudoxids)	DCM	4.0±1.0 ^ab^	4.4±1.2 ^b^	(both stages)	DCM	0.4±0.3 ^ab^	0.0
	BL	5.7±1.2 ^a^	0.3±0.2 ^b^		BL	0.0 ^b^	0.0
	F	**4.14**	**39.01**		F	**4.045**	3.00
	p	**0.0249**	**<0.0001**		p	**0.0268**	0.0635
*A*. *tetragona*	UML	3.2±0.8 ^a^	6.9±1.0 ^a^	*B*. *bassensis*	UML	0.6±0.3	1.0±0.4 ^a^
(polygastrics)	DCM	1.1±0.4 ^b^	1.7±0.4 ^b^	(both stages)	DCM	0.6±0.2	0.0 ^b^
	BL	1.4±0.5 ^ab^	0.0 ^b^		BL	0.0	0.0 ^b^
	F	**3.543**	**33.71**		F	2.533	**6.061**
	p	**0.0404**	**<0.0001**		p	0.0947	**0.0057**
*D*. *bojani*	UML	9.5±1.7^a^	8.9±1.4 ^a^	*C*. *appendiculata*	UML	1.5±0.4 ^a^	0.9±0.4 ^a^
(eudoxids)	DCM	7.8±2.1^a^	0.1±0.1 ^b^	(eudoxids)	DCM	0.3±0.2 ^b^	0.1±0.1 ^ab^
	BL	1.7±0.9^b^	0.1±0.1 ^b^		BL	0.0 ^b^	0.0 ^b^
	F	**6.050**	**36.04**		F	**8.157**	**4.674**
	p	**0.0058**	**<0.0001**		p	**0.0013**	**0.0163**
*D*. *bojani*	UML	3.7±0.9	3.8±0.7 ^a^	*C*. *appendiculata*	UML	0.1±0.1	0.2±0.1
(polygastrics)	DCM	3.1±0.6	0.0 ^b^	(polygastrics)	DCM	0.3±0.2	0.4±0.2
	BL	1.4±0.6	0.1±0.1 ^b^		BL	0.1±0.1	0.1±0.1
	F	2.958	**27.23**		F	0.3604	0.6494
	p	0.0658	**<0.0001**		p	0.7820	0.5289
*A*. *eschscholtzii*	UML	1.7±0.5 ^a^	2.9±0.8 ^a^	*Beroe* sp.	UML	0.2±0.1	2.8±0.7 ^a^
(eudoxids)	DCM	0.1±0.1 ^b^	0.3±0.3 ^b^		DCM	0.4±0.3	1.0±0.4 ^ab^
	BL	0.0 ^b^	0.0 ^b^		BL	1.9±0.9	0.4±0.4 ^b^
	F	**10.68**	**11.12**		F	2.542	**4.882**
	p	**0.0003**	**0.0002**		p	0.0940	**0.0139**
*D*. *nationalis*	UML	9.9±2.2	5.7±1.5 ^a^	*D*. *gegenbauri*	UML	3.3±1.2 ^b^	4.0±1.0 ^a^
(phorozooids)	DCM	13.0±3.8	2.7±1.4 ^ab^	(phorozooids)	DCM	15.4±5.4 ^a^	2.7±0.9 ^ab^
	BL	7.4±4.1	0.1±0.1 ^b^		BL	5.1±2.5 ^b^	0.5±0.2 ^b^
	F	0.6567	**5.399**		F	**3.429**	**5.235**
	p	0.5252	**0.0094**		p	**0.0443-**	**0.0106**
*D*. *nationalis*	UML	42.9±11.9 ^a^	28.0±9.1 ^a^	*D*. *gegenbauri*	UML	1.6±0.7	1.1±0.3 ^a^
(gonozooids)	DCM	16.0±6.0 ^ab^	1.5±0.6 ^b^	(gonozooids)	DCM	1.3±0.5	0.1±0.1 ^b^
	BL	3.4±1.8 ^b^	0.1±0.1 ^b^		BL	0.6±0.3	0.0 ^b^
	F	**6.734**	**8.863**		F	0.8206	**11.11**
	p	**0.0035**	**0.0008**		p	0.4489	**0.0002**
*D*. *nationalis*	UML	3.2±0.8 ^ab^	4.0±0.8 ^a^	*D*. *gegenbauri*	UML	2.8±0.6 ^ab^	4.2±0.9 ^a^
(old nurses)	DCM	5.6±1.3 ^a^	0.6±0.4 ^b^	(old nurses)	DCM	3.8±0.7 ^a^	2.1±0.5 ^ab^
	BL	1.2±0.5 ^b^	0.1±0.1 ^b^		BL	0.8±0.5 ^b^	0.3±0.2 ^b^
	F	**5.711**	**17.46**		F	**6.115**	**10.09**
	p	**0.0074**	**<0.0001**		p	**0.0055**	**0.0004**
*T*. *democratica*	UML	10.3±4.4	15.3±4.2 ^a^	*S*. *fusiformis*	UML	3.0±1.6	3.7±1.8
(aggregates)	DCM	18.1±5.6	2.4±.10 ^b^	(aggregates)	DCM	3.4±1.8	1.5±0.8
	BL	19.0±11.5	0.0 ^b^		BL	4.2±.21	0.5±0.2
	F	0.3764	**10.92**		F	0.1046	2.118
	p	0.6892	**0.0002**		P	0.9010	0.1363
*T*. *democratica*	UML	0.5±0.2	0.3±0.2	*S*. *fusiformis*	UML	1.5±0.5	1.2±0.6
(solitaries)	DCM	2.0±0.6	0.0	(solitaries)	DCM	0.7±0.3	0.8±0.3
	BL	2.3±1.2	0.1±0.1		BL	2.3±1.2	0.0
	F	1.347	1.477		F	1.013	2.483
	p	0.2738	0.2429		p	0.3742	0.0989

The abundance of gelatinous zooplankton changed considerably during the study, and was highest during the first morning (Fig 5B). Nevertheless the general vertical structure was always similar. These zooplankters were more widely distributed through the water column during the day and more aggregated in the UML at night (Fig 5B; Table 2). Abundances were always lower in the BL, decreasing to a mean of <6 ind 10 m^-3^ at night. The absence of significant vertical differences when all diurnal samples were included (Table 2) is due to the higher abundance found in the first morning (when the mean was ~305 ind 10 m^-3^ in the BL) which caused noise in the analysis (as evidenced by the much larger SD in the diurnal BL compared to the nocturnal BL); when the data from this occasion were excluded, the diurnal abundances were significantly smaller (t test, p<0.05) in the BL than in the other strata.

**Fig 6:**
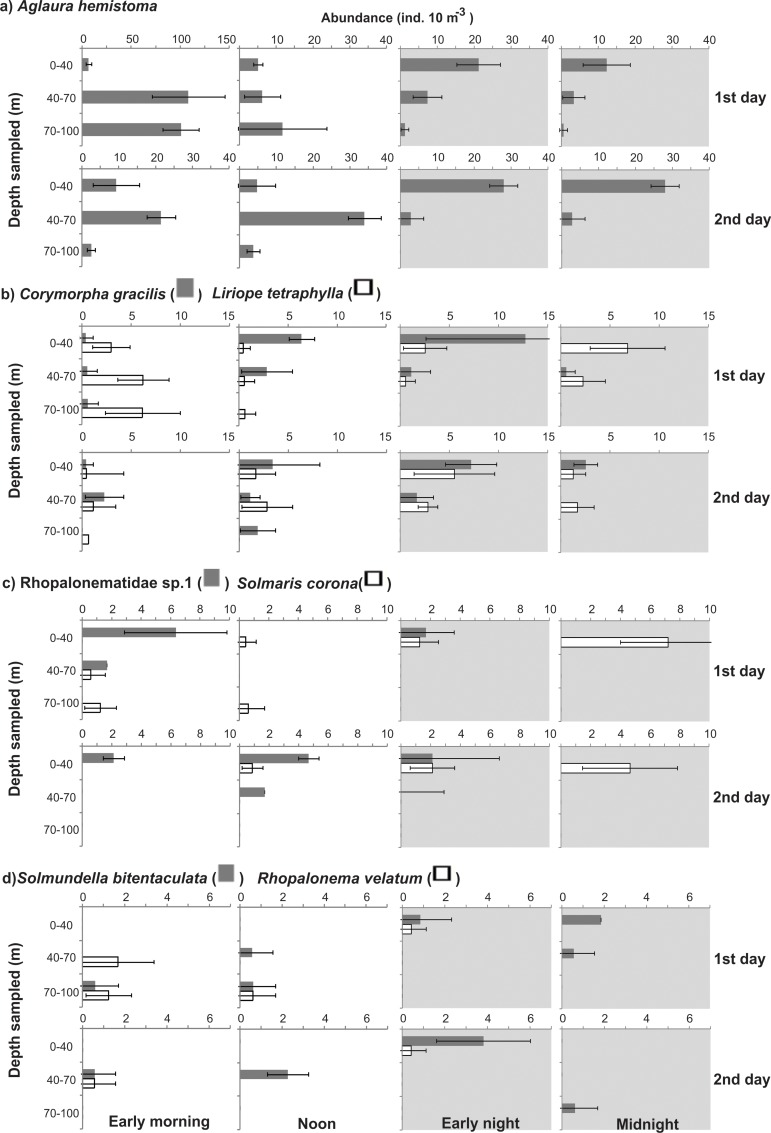
Vertical distribution of most abundant medusae. Shadows indicate nocturnal periods. Values are shown as average ± standard deviation. Notice different scales.

**Fig 7:**
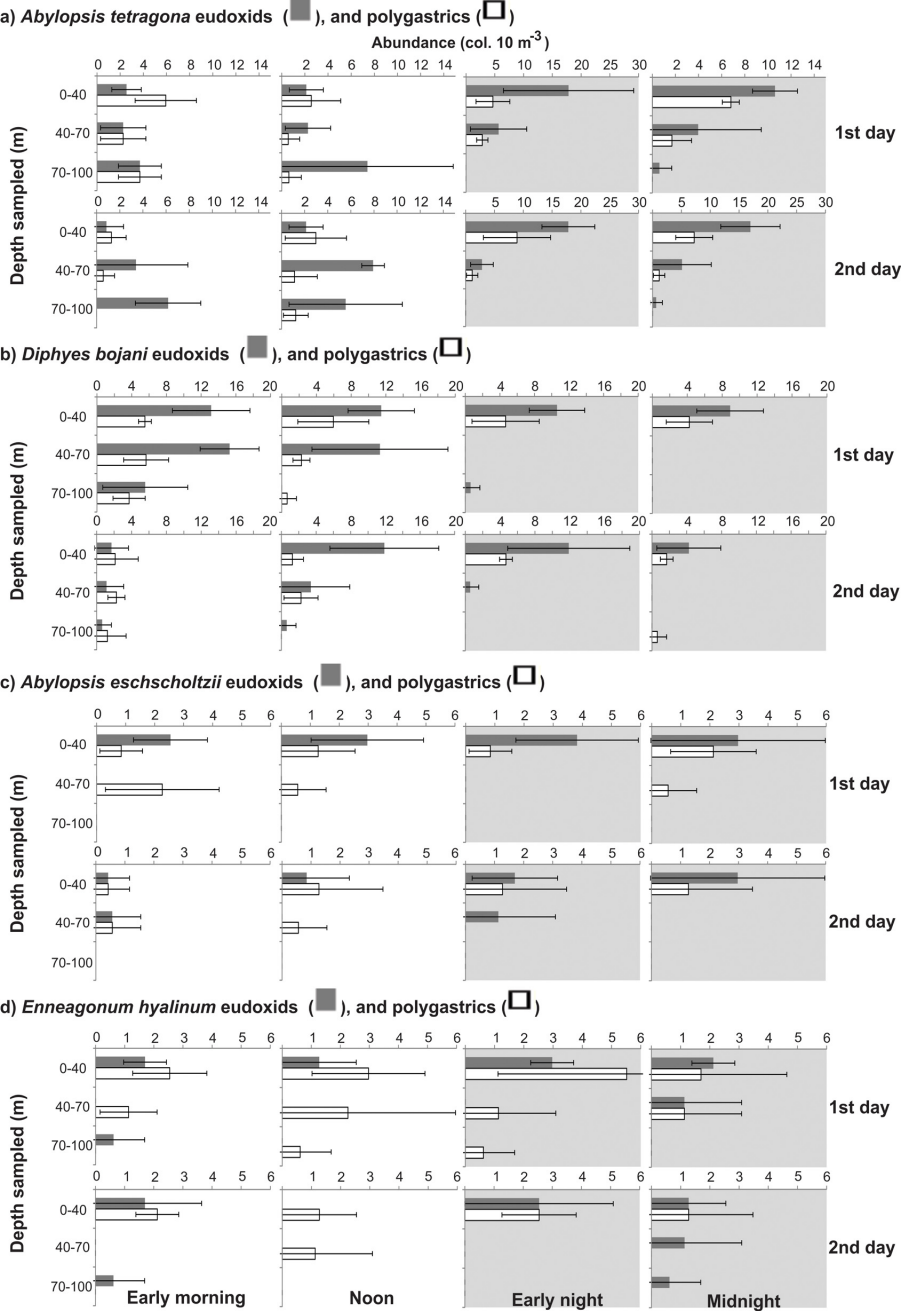
Vertical distribution of most abundant siphonophores. Shadows indicate nocturnal periods. Values are shown as average ± standard deviation. Notice different scales.

**Fig 8:**
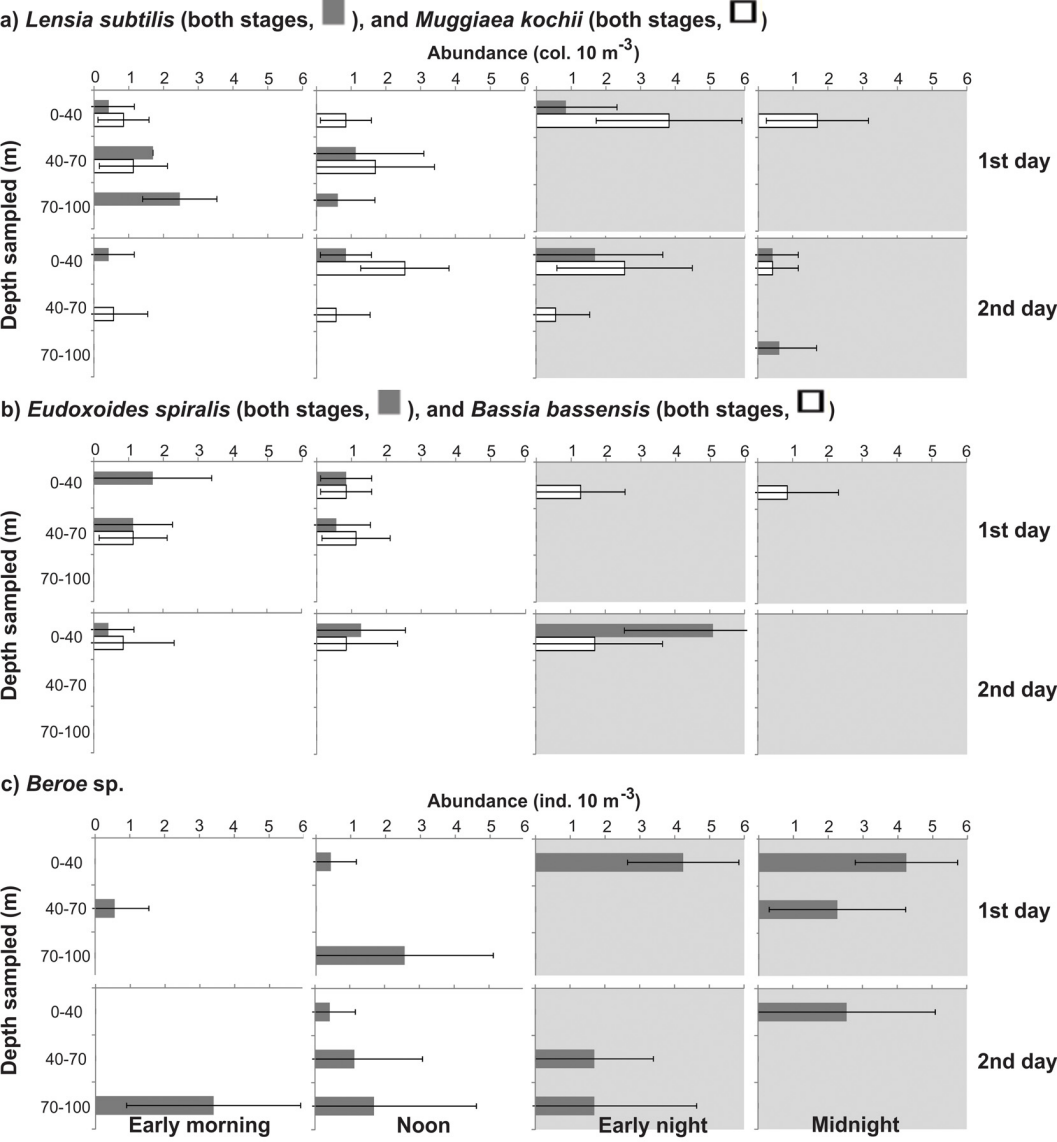
Vertical distribution of other common siphonophores and the most abundant ctenophore. Shadows indicate nocturnal periods. Values are shown as average ± standard deviation. Notice different scales.

**Fig 9:**
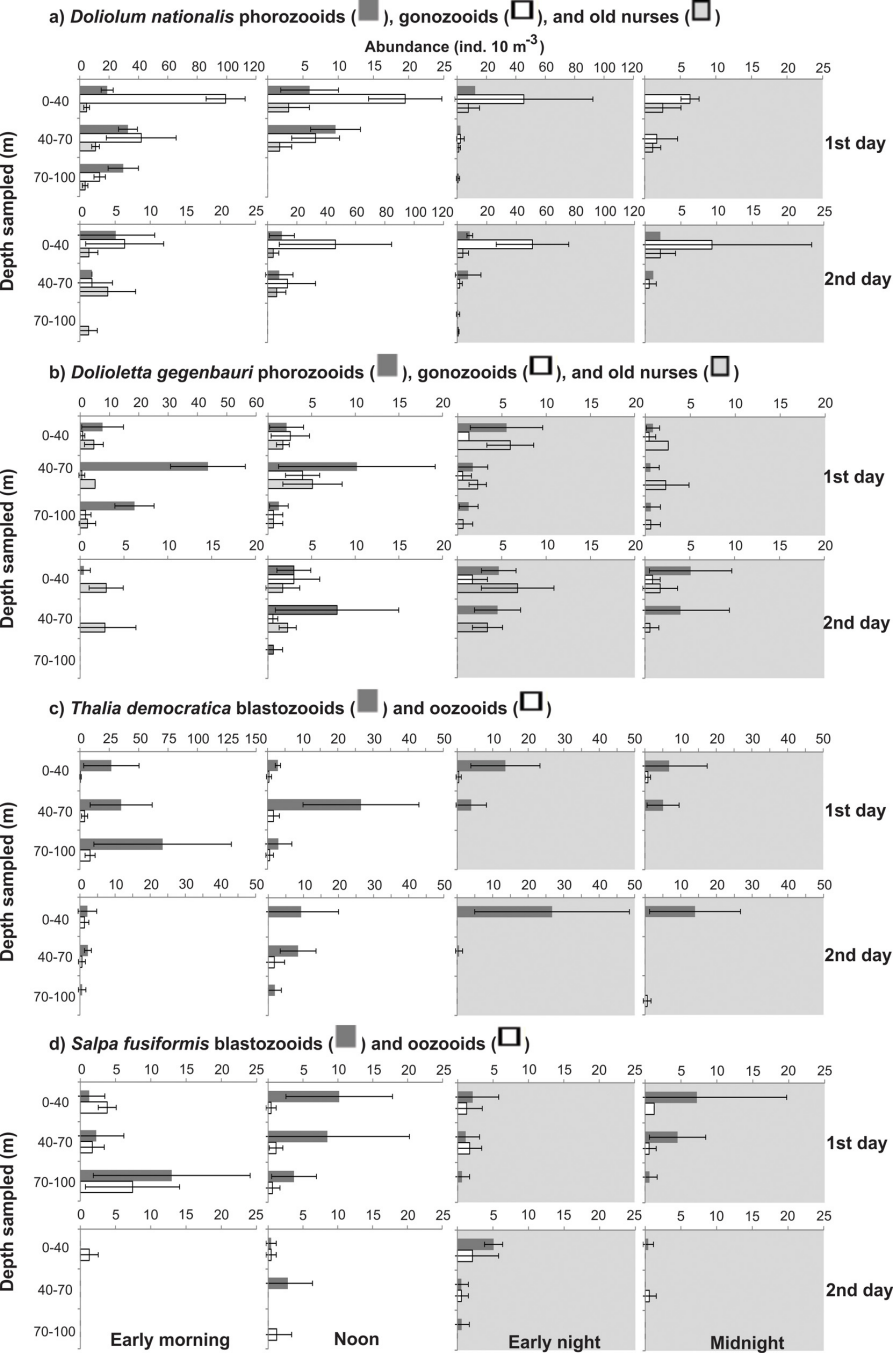
Vertical distribution of most abundant thaliaceans. Shadows indicate nocturnal periods. Values are shown as average ± standard deviation. Notice different scales.

**Fig 10:**
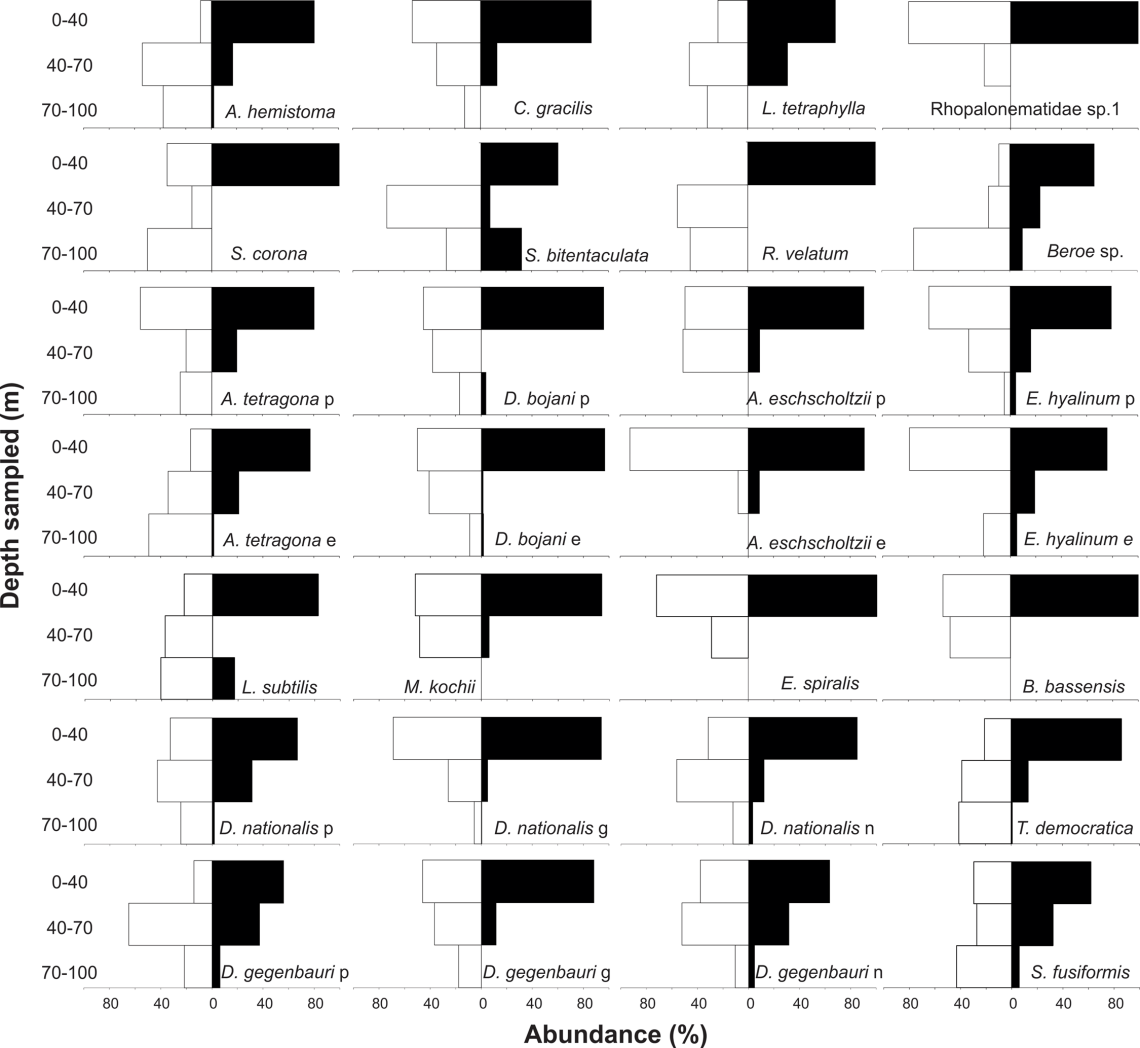
Summary of the diurnal and nocturnal vertical distribution of gelatinous zooplankton from South Brazilian Bight. Data is presented as percentage of the total abundance (ind. 10 m^-3^) found in the water column during diurnal (open bars) and nocturnal (black bars) samplings combining both days. e = eudoxids, p = polygastrics, g = gonozooids, p = phorozooids, n = old nurses, whenever not indicated developmental stages were pooled due to similar distribution.

The abundances of most taxa differed significantly (ANOVA, p<0.05) in the different depth strata, in both diurnal and nocturnal samples (Table 2). Most species were always less abundant in the BL, independently of the period of the day; and taxa such as Rhopalonematidae sp.1, *Abylopsis eschscholtzii*, *Muggiaea kochii*, *Eudoxoides spiralis* and *Bassia bassensis* were completely absent from this layer. A general tendency toward partial migrations was clear. Most species aggregated in the UML at night, and part of the population (a minor part in many cases) descended during daytime. The proportion of the population descending and the stratum that they reached varied with the different taxa and developmental stages (Figs 6–10; Table 2). Most did not reach the BL in considerable proportions, descending mostly to the DCM during the day. In a few taxa such as *Beroe* sp. and *A*. *tetragona* eudoxids, a considerable part of the population clearly crossed the thermocline on both days; while others such as *A*. *hemistoma*, *Liriope tetraphylla*, *Rhopalonema velatum*, *Lensia subtilis*, *Thalia democratica* and *Salpa fusiformis* were mostly in the BL in the first morning and/or noon, but did not repeat this on the second day, when they descended to the DCM (Figs 6–10).

Grouping data from both days, the pattern of nocturnal aggregation in the UML is clear. In species such as *Beroe* sp., *A*. *hemistoma*, *A*. *tetragona* eudoxids and *Dolioletta gegenbauri* phorozooids, most of the population (>80%) was deeper than the UML during daylight, with significantly different diurnal and nocturnal weighted mean depths (t test, p<0.05; S1–S4 Tables). In others such as *L*. *tetraphylla*, *D*. *bojani*, *M*. *kochii*, *S*. *fusiformis*, *T*. *democratica*, and old nurses of both doliolids, a considerable part of the population (>40%) was migrating (Fig 10), still resulting in statistically significant differences between the diurnal and nocturnal weighted mean depths. For most other taxa, only a small part of the population was extending their vertical position during daytime; and for *A*. *eschscholtzii* eudoxids, *Enneagonum hyalinum*, and Rhopalonematidae sp.1 the proportion of the population in the UML in the diurnal and nocturnal periods was nearly the same (Fig 10).

### Other zooplankton groups

**Fig 11:**
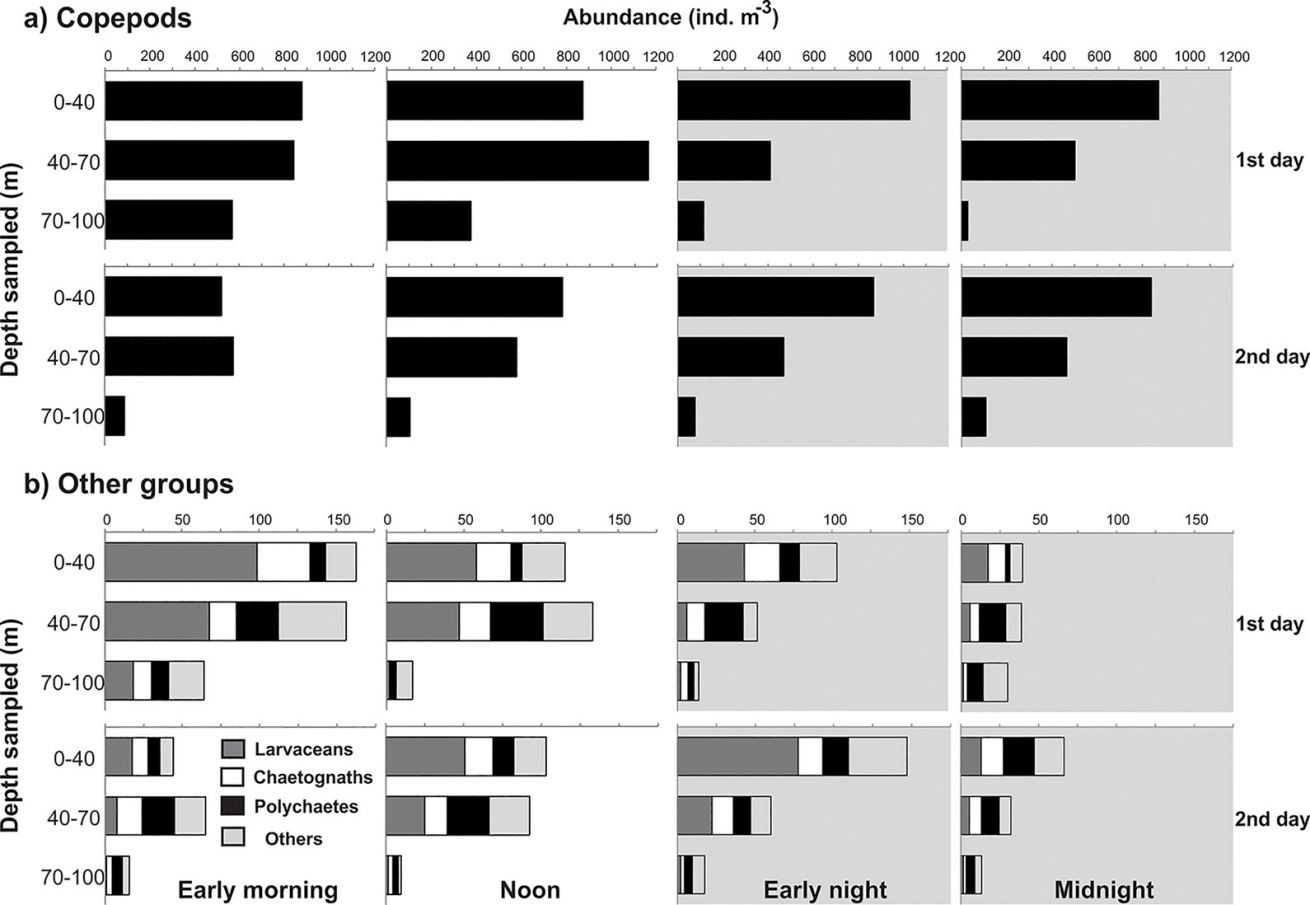
Vertical distribution of copepods and other zooplankton groups. Abundances were estimated from the first series of the triplicate samples and were based on counts of whole samples (larvaceans and chaetognaths) or 1/4 to 1/32 aliquots (all other taxa). Shadows indicate nocturnal periods. Notice different scales.

Copepods were the most abundant, comprising 89.2% of the zooplankton individuals (excluded the gelatinous groups detailed above), followed by larvaceans (4.0%), polychaetes (2.1%; mostly terebellid larvae), and chaetognaths (1.9%). Other taxa totaled <3% and were represented by typical zooplankton taxa of shelf waters such as mero- and holoplanktonic molluscs, cladocerans, amphipods, euphausiaceans, ostracods, decapods, stomatopods and ichthyoplankton. The most abundant copepods were *Ctenocalanus vanus* (31.6% of copepod individuals), *Oncea waldemari* (23.8%), and *Oithona plumifera* (11.1%). In general, zooplankton was sparse in the BL, independently of the period of the day. Their highest densities were recorded during early morning and noon of the first day. Copepods (Fig 11A) tended to be similarly distributed in the two upper strata during the day and more aggregated in the UML during the night. Larvaceans and chaetognaths were typically more abundant in the UML independently of the period of the day, and almost absent from the BL; however their abundance in the DCM tended to increase during diurnal samples (Fig 11B). Polychaetes had a different pattern; they were mostly found in the DCM, except at night on the second day, when they were more concentrated in the UML.

### Relationship to environmental factors and food availability

**Fig 12:**
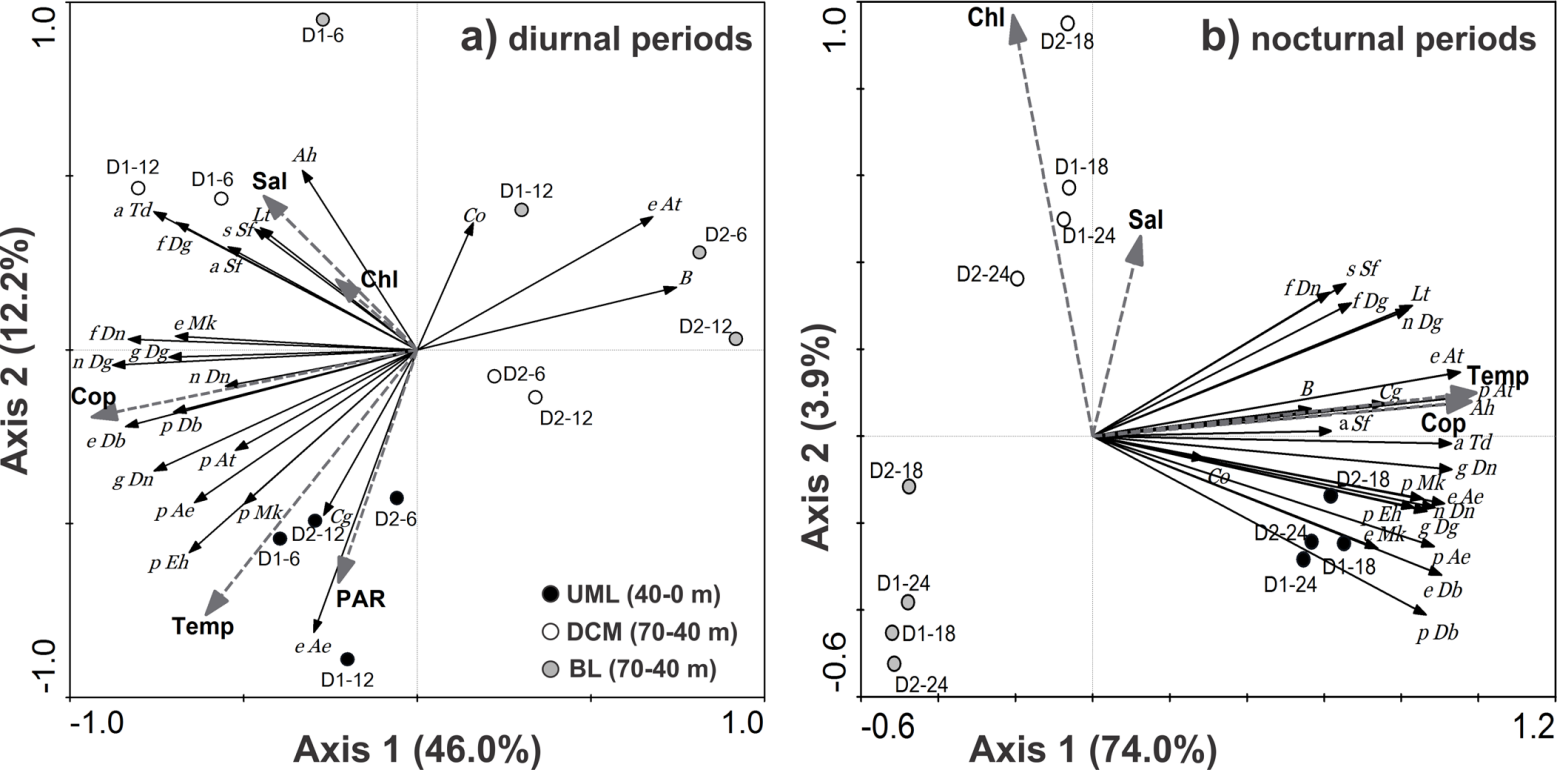
Ordination diagrams of the Redundancy Analysis. Graphs show the relationship of species/developmental stages and biotic and abiotic explanatory variables during diurnal (a) and nocturnal (b) periods showing the first and second canonical axes. Species are shown as black continuous vectors and explanatory variables as dotted grey vectors. Samples are shown as circles with colors changing according to the depth strata (see legend) and named according to the first or second day sampled (D1 or D2 respectively) and the time of sampling. The percentage of the species data variation explained by each environmental axis is shown in parentheses. **Environmental variables codes:** Chl = chlorophyll-*a*, Cop = copepods, PAR = photosynthetically active radiation, Sal = salinity, Temp = temperature. **Species codes:** Ae = *Abylopsis eschscholtzii*, Ah = *Aglaura hemistoma*, At = *Abylopsis tetragona*, B = *Beroe* sp., Cg = *Corymorpha gracilis*, Co = *Cordagalma ordinatum*, Db = *Diphyes bojani*, Dg = *Dolioletta gegenbauri*, Dn = *Doliolum nationalis*, Eh = *Enneagonum hyalinum*, Lt = *Liriope tetraphylla*, Mk = *Muggiaea kochii*, Sf = *Salpa fusiformis*, Td = *Thalia democratica*. The first letter before the species name refer to the life cycle stage of calicophorans (e = eudoxids, p = polygastrics) and thaliaceans (a = aggregate zooids, f = phorozooids, g = gonozooids, n = old nurses, s = solitary zooids). UML = upper mixed layer; DCM = deep chlorophyll-*a* maximum layer; BL = bottom layer.

Both diurnal and nocturnal samples were clearly separated according to depth strata in the ordination diagram (Fig 12A and 12B). The first four canonical axes explained 68.5% of the diurnal species variance (Table 3). The first axis explained 46% and was mostly negatively related to copepods and temperature; the second axis explained a further 12% and was mostly negatively related to temperature and PAR (Table 3; Fig 12A). The third and fourth axes were mostly related to salinity, PAR and chlorophyll-*a*, and together explained an additional ~10% (Table 3). Most species and developmental stages were negatively related to the first axis, and therefore positively related mostly to copepod abundance and temperature, except for *Beroe* sp. and *A*. *tetragona* eudoxids, which were positively related to the first canonical axis. *Corymorpha gracilis*, *A*. *eschscholtzii*, and *M*. *kochii* and *E*. *hyalinum* polygastrics were positively related to temperature and PAR, while *A*. *hemistoma*, *L*. *tetraphylla*, *S*. *fusiformis*, *T*. *democratica*, and *D*. *gegenbauri* phorozooids were more related to salinity and chlorophyll-*a* (Fig 12A).

**Table 3:** Summary of the Redundancy Analysis. This analysis was performed between the 23 dominant gelatinous zooplankton taxa/developmental stage and the selected explanatory variables during diurnal and nocturnal periods.

RDA Summary	Axis 1	Axis 2	Axis 3	Axis 4
**Diurnal**				
Eigenvalues	0.460	0.122	0.065	0.037
Species-explanatory correlations	0.865	0.923	0.926	0.958
% of variance explained (species data)	46.0	12.2	6.5	3.8
Accumulated variance (%)
Of species data	46.0	58.2	64.7	68.5
Of species-explanatory relationship	66.2	83.6	93.1	98.4
Correlations of explanatory variables				
Temperature (°C)	0.6085	-0.7626	0.1961	0.0565
Salinity	0.4422	0.4422	0.5028	-0.5867
PAR	0.2263	-0.6686	-0.5115	-0.2557
Chlorophyll-a (mg m^-3^)	0.233	0.2017	0.3299	-0.7426
Copepods (ind. m^-3^)	0.9368	-0.1932	-0.1180	-0.1755
**Nocturnal**				
Eigenvalues	0.740	0.039	0.018	0.006
Species-explanatory correlations	0.978	0.913	0.728	0.680
% of variance explained (species data)	74.0	3.9	1.9	0.6
Accumulated variance (%)				
Of species data	74.0	77.9	79.8	80.4
Of species-explanatory relationship	92.0	96.9	99.2	100.0
Correlations of explanatory variables				
Temperature (°C)	0.993	0.1001	0.0113	-0.0490
Salinity	0.1243	0.4594	0.6839	0.5529
Chlorophyll-a (mg m^-3^)	0.2061	0.9690	-0.1359	0.0037
Copepods (ind. m^-3^)	0.9812	0.0799	-0.0303	0.1728

Temperature and copepod abundance were highly auto-correlated and were the explanatory variables that most influenced the first axis, which accounted for 74% of the nocturnal species variance. All species and developmental stages tended to increase in the UML at night, and therefore were closely related to temperature and copepod abundance (Fig 12B). The third and fourth axes were related to salinity, but together explained only an additional 2.5% of the nocturnal data variance (Table 3).

## Discussion

Short-term repetitive sampling such as the regime used here is mostly lacking in the literature on Brazilian marine zooplankton [38]. This kind of sampling has the advantage of suggesting relationships between zooplankton and potentially explanatory variables on a temporal scale that is more compatible with the ecological and behavioral processes affecting DVM [20]. Based on the short sampling scale and the relatively high sampling effort (i.e. triplicate hauls over two consecutive days), we consider that the assemblage structure and the daily dynamics of vertical occupation are well represented for the study site. Our data are limited to a single cruise and therefore do not address possible seasonal variations; however, large seasonal variations in the oceanographic configuration and in the zooplankton abundance and assemblage structure are not expected in offshore areas of the South Brazilian Bight [23–25, 39]. The depth levels of the layers sampled were chosen according to the oceanographic structure of the water column, and do not allow the depiction of possible smaller-scale patterns such as DVM within the same water mass [2, 19, 40].

### Oceanographic structure

The relatively constant oceanographic conditions over the short sampling period indicate that the system was stable. The three-layered oceanographic structure observed, with warm TW influence in the UML, an intermediate thermocline and associated DCM, and the colder BL under SACW influence is typical of outer-shelf waters in the South Brazilian Bight year-round [23–25]. The lower salinities (35–36.0) in the upper layer, along with the presence of typical taxa of the Coastal Water such as *Obelia* spp., actinula larvae, *L*. *tetraphylla*, and *M*. *kochii* [24, 25, 39, 41], also suggest the influence of this water mass. This probably results from wind-driven offshore advection of Coastal Water, which forms a surface haline front over the shelf that separates Tropical Water from Coastal Water and may reach over 100 km offshore [23–25, 39]. In this study we sampled at a single station and therefore cannot precisely locate the position of the front; however, the salinities lower than 36 in the upper 20 m indicate that our station was probably close to it.

### Vertical dynamics of gelatinous zooplankton

#### Influence of physical stratification of the water column

Differences in the depth strata accounted for most of the assemblage variability (Table 1), and the vertical stratification of the assemblage clearly followed the physical stratification of the water column: most gelatinous zooplankton were absent or little abundant in the BL independently of the period of the day, and temperature had a high explanatory power for both the diurnal and nocturnal data-sets. Even among migratory taxa, only a few such as *Beroe* sp., *A*. *tetragona* eudoxids, *A*. *hemistoma*, *T*. *democratica* and *S*. *fusiformis* crossed the thermocline. These observations suggest that the cold SACW restricted the vertical occupation of most species, which typically inhabit epipelagic warm waters [7, 8, 26, 39, 40], emphasizing the role of the physical vertical stratification of the water column in shaping the assemblages of gelatinous zooplankton. A previous investigation of the vertical distribution of gelatinous zooplankton in the South Brazilian Bight showed the seasonal influence of the SACW, with overall low species richness and abundance in the BL when it is influenced by this water mass [39]. The present data agree with this observation and offer additional details on vertical patterns of the gelatinous zooplankton, owing to the finer scale of temporal sampling and to the analysis of other gelatinous taxa whose distributional patterns are largely understudied and therefore unknown in the southwestern Atlantic (i.e. ctenophores and thaliaceans).

The dominant hydromedusae in the present study, *A*. *hemistoma*, *L*. *tetraphylla*, and *C*. *gracilis*, are vertically limited by physical stratification of the water column in many ecosystems [2, 8, 20], although *A*. *hemistoma* was performing DVM through the thermocline in the present study (Figs 6A and 10). Likewise, most calicophorans in stratified systems such as the Arabian Sea [40, 42, 43], Benguela Current [7] and Humboldt Current System [30, 44] are commonly vertically restricted by the thermocline, and typically tend to remain above it. The vertical distribution of the most abundant siphonophore in this study, *A*. *tetragona*, can be quite contrasting in different environments. It typically inhabits the superficial layers in both stratified and non-stratified situations in oceanic and shelf regions of the Mediterranean [45–48], North and South Atlantic [7, 49, 50], and eastern Indian Ocean [40, 43], but it also may occur deeper [52, 53]; moreover, nocturnal ascension may occur ([18, 43, 51, 52], present study) or not [7, 45], and the thermocline may [7] or may not [42] restrict its vertical distribution. In the present study, polygastrics were apparently restricted by the thermocline, mostly remaining above it independently of the period of the day; while eudoxids were undergoing DVM through the thermocline. Similarly, *D*. *bojani* typically remains above the thermocline ([7, 40], present study) but also may cross it [42].

Differently from the present observations, *Beroe* spp., *Mnemiopsis leidyi* and *Pleurobrachia* spp., the best-studied ctenophores worldwide, do not migrate through the physical stratification, typically remaining above or below the pycnocline/thermocline independently of the period of the day [19, 53–56]. Diel vertical migration has been reported for ctenophores in vertically homogeneous water columns: *Beroe cucumis* in the northeastern Atlantic migrates upward by night, from 250–600 to 100 m, in a vertically homogeneous situation [57]; *B*. *ovata* in the Black Sea may undergo a short DVM in the homogeneous upper 20 m depth [19]; and a similar trend with a 10-m DVM occurs for *M*. *leidyi* populations in the Baltic Sea [58].

Although there are few studies on doliolid DVM, the pattern observed here, similar for both species, concords with previous observations that these organisms essentially inhabit superficial warm waters [31, 59] and typically occur in or above the thermocline [60, 61]. *Doliolum denticulatum*, a species closely related to those reported here, is limited by temperatures lower than 15°C [62]. Such temperatures were observed in the lower 20 m of the water column in the present investigation, and may have been a factor limiting the distribution of *D*. *nationalis* and *D*. *gegenbauri*, both of which were virtually absent from the BL.

The vertical distribution pattern observed for both salps was variable, but they were clearly able to cross the thermocline and occupy the BL. This observation contrasts with previous reports of *T*. *democratica*, which typically remains in the upper layers independently of the period of the day, in different ecosystems such as the Kuroshio Current [63], southern Brazil [21], South Africa [64], northwestern Mediterranean [51, 52, 65] and Taiwan [31]. Species of the genus *Salpa* are strong swimmers and typically extensive migrators [66], and *S*. *fusiformis* is insensitive to low temperatures (<10°C) [67], a physiological adaptation that allows it to withstand temperature changes during vertical migrations. *S*. *fusiformis* may remain mostly above the thermocline in the East Sea off Korea [68], or with solitary individuals mostly below it in the Kuroshio Current [63]. Moreover, it can be vertically widespread during daylight and more aggregated near the surface at night [52, present study], or have a discontinuous bimodal distribution [69]. Distinct patterns were also observed in the present study, where most of the population was in the BL during the first morning and then in the UML by noon (Fig 9D), suggesting that the patterns of vertical distribution of this salp may not be related to the diel cycle.

#### The diel cycle and the vertical migrations

The vertical structure of the assemblage was not maintained throughout the daily cycle, as suggested by the statistical significance of the interaction between the factors “depth stratum” and “period of the day” in the PERMANOVA (Table 1). The results of the cluster analysis also support this view. As a result of the migrations, (a) the nocturnal samples from the BL were quite different from all others (<25% of similarity), with the virtual absence of almost all gelatinous zooplankters and therefore low species richness (0–6 spp.) and abundance (<11 ind. 10 m^-3^); (b) the DCM assemblages were more similar either to the UML or to the BL depending on the period of the day. The diurnal downward migrations, together with the influence of stratification on most species (see above), increased the similarity between the diurnal DCM and both the diurnal and nocturnal UML samples (group B). On the other hand, the nocturnal upward migration of most taxa resulted in a similarity between the diurnal BL assemblages and the nocturnal DCM assemblages (group C).

Light is commonly recognized as an important signal for zooplankton DVM [1, 5, 6, 9]. Accordingly, the migrations recorded here clearly followed the light/dark cycle, and the light intensity seems to have influenced the vertical patterns of many species. During the first day, the light intensity was higher (Fig 3B) and most taxa as well as the total abundance of gelatinous zooplankters showed greater vertical changes than on the following day. Most gelatinous zooplankters react to changes in light intensity, and the influence of light on their behavior and vertical distribution has been shown for all main groups, including cnidarians [20, 70–72], ctenophores [9, 19, 58, 73], and thaliaceans [52, 74]. Although many of these species do not have ocelli or other organized photosensitive structures, cnidarian neurons may be directly stimulated by light [74, 75] and extraocular photosensitivity is widespread in invertebrates, occurring in most if not all groups of gelatinous zooplankton [75, 76].

However, if light were the main factor driving the migrations reported here, it would be expected to affect all individuals of a given species similarly, leading to downward migrations of whole populations, which was not the case. The results obtained here for virtually all species and developmental stages clearly show that only a part of the populations was performing diurnal downward migrations. This general pattern encompassed phylogenetically and ecologically distinct gelatinous taxa, and can be interpreted as a behavioral convergence providing an adaptive advantage. Indeed, migrations performed by only a part of the population emerge as an optimal evolutionary behavior in theoretical models [77] and probably are very common in nature due to individual and environmental variability, particularly when food availability is low [1, 5, 78] as is the case for the typically oligotrophic offshore waters studied here. Differences at the individual level have been emphasized, and diverse behaviors may simultaneously occur in a given population, suggesting that the DVM patterns of zooplankton (and also phytoplankton; see [79]) may be much more complex than formerly thought [10, 80–83].

The overall DVM pattern found here fits well with the model of the “hunger-satiation” hypothesis, a strategy to maximize feeding and minimize the chances of being predated (reviewed by Pearre [5]). Increasing experimental, field and theoretical evidence [5, 10, 82, 84, 85] suggests that whether vertical migration occurs or not may reflect a balance between the two main conflicting factors that affect the survival of each individual: food capture and avoiding the risk of being predated [84]. Thus, individuals tend to spend the least possible time feeding at shallow depths, where they are at greater risk of being predated; and move down when they become satiated [5]. These feeding incursions become optimal if they occur during the night, when visual predation is more difficult. The station where the present samples were taken is mostly oligotrophic, as is typical of the South Brazilian Bight outer shelf, with relatively low concentrations of both phytoplankton and zooplankton year-round [23]. In situations with low food availability (and/or high competition), individuals would tend to remain feeding near the surface until they consumed enough food to stimulate the beginning of the DVM. In these cases, the risk of being eaten by predators is lower than that of dying from inanition, and individuals may remain near the surface during daylight if they are unable to become satiated during the night [84–86].

Thaliaceans are typically herbivorous [59, 64], and therefore one would expect them to be mostly associated with the DCM where chlorophyll-*a* concentrations are higher. However, they also may use other sources of food not measured in this study, such as auto- and heterotrophic microbes of the pico- and nanoplankton size classes [15, 59], which have been reported to be more abundant in the UML in oligotrophic waters offshore of Brazil [87]. Moreover, the salp pharynx is not adapted to high particle concentrations and may clog permanently [59], a constraint that has been invoked to explain the generally low relationship between their vertical distribution and DCMs [64].

Although an endogenous circadian rhythm may occur in many planktonic taxa [6], the migration rhythm of many gelatinous zooplankters is not intrinsic [70, 71] and therefore their vertical position can be adjusted according to many external factors, such as hydrography, trophic interactions, nutritional condition, luminosity and others. The complex trade-off between these biotic and abiotic factors may sometimes result in contrasting distributions of the same species or developmental stage in different situations. This may be particularly true for widely distributed species that may occur over a wide range of environmental conditions, such as most of the dominant species here, *A*. *hemistoma*, *Beroe* sp. *A*. *tetragona*, *D*. *bojani*, *T*. *democratica*, and *S*. *fusiformis*.

In spite of the homogeneous hydrographic conditions found throughout our study, internal waves associated with tidal cycles are an important physical factor that could possibly explain the vertical displacement of specific water layers, and hence the short-term differences in the vertical distribution of planktonic organisms. Although most studies on the issue were conducted in lakes [88–90], in marine environments a few studies also have reported the effects of internal waves on zooplankton vertical dynamics, usually associated with tidal currents progressing from deep to shallow waters [91]. Internal waves are conspicuous in the South Brazilian Bight, with the potential to displace water-column isotherms up to 28 m [25, 92]. In the present study, the 25 isopycnal was positioned around 30 m depth at 06:00 of the first sampling day, and ascended to 17 m by 18:00 (Fig 2), perhaps due to internal waves associated with tidal cycles and which may have influenced the observed zooplankton vertical distribution to some unknown extent.

#### Ontogenetic differences

Differences in the vertical distribution of the different life-cycle stages were obvious for the siphonophores *A*. *tetragona*, *A*. *eschscholtzii* and *Chellophyes appendiculata* as well as for both doliolid species, emphasizing the importance of separately analyzing the different life-cycle stages. While ontogenetic shifts in the vertical patterns are well known for many crustaceans, with numerous descriptions available in the literature (e.g. [1, 26, 32, 93]), this is not the case for most gelatinous taxa. Among the few such reports for siphonophores are those on *Chuniphyes multidentata*, *Muggiaea bargmannae* and *Nectopyramis spinosa*, whose eudoxids are always deeper than the polygastrics [50, 94, 95]; and on the Mediterranean *A*. *tetragona*, whose polygastrics but apparently not eudoxids are found below the seasonal thermocline [46]. The reasons leading to such differences are difficult to establish, and the desirability of reaching water of the ideal temperature for sexual reproduction has been suggested to explain the differences in *M*. *bargmannae* from the Weddell Sea [95].

The ontogenetic differences observed for both doliolids concord with previous observations. Gonozooids typically inhabit more superficial depths than oozooids, which may make a small nocturnal ascent [31, 60]. The higher abundance of gonozooids in the UML observed here is possibly related to the warm temperature in this stratum, which is an important factor in the growth, reproduction and longevity of this stage [96, 97]. The highest abundance of phorozooids over the thermocline, where chlorophyll-*a* peaks occur, may be associated with food availability, which is more important for asexual reproduction than is temperature [97].

## Conclusions

The present observations showed that the assemblages of gelatinous zooplankton in oligotrophic subtropical ecosystems such as the offshore South Brazilian Bight typically perform partial DVM, aggregating in the UML by night, with a variable part of the populations (a minor part in many cases) descending during daylight to either the DCM or the BL, depending on the species and developmental stage. It is difficult to ascertain the direct causal factors leading to these distributions at the specific level, particularly because in field studies it is difficult to disentangle proximate and ultimate causes [81] as well as the many different covarying factors, in addition to the slight knowledge of the biology and physiology of most gelatinous species. In any case, the overall drivers of the assemblage could be clearly inferred.

Two main general patterns of daily vertical dynamics were clear, underlining the role of physical (i) and biological (ii) drivers in structuring the gelatinous zooplankton assemblages. (i) Most species typically inhabit epipelagic warm water and were therefore absent or little abundant in the bottom layer under the influence of the cold SACW, emphasizing the important effect of the physical vertical stratification of the water column. (ii) A general tendency of partial migrations with populations aggregated in the UML during the night, and a variable part of them descending during daylight. While these migrations clearly followed the diel light/dark cycle, not all individuals behaved similarly, in a pattern that can be explained by the hunger-satiation hypothesis; only well-fed individuals would migrate downward during the day. Therefore, we suggest that the diel patterns of vertical occupation of the gelatinous zooplankton assemblage found here are the result of a trade-off among predator avoidance, food availability, and ideal hydrographic conditions for a particular species or developmental stage.

## Supporting Information

S1 Dataset The samples are labeled as follow: D1 or D2 refers to the first and second sampling day; the subsequent number (6, 12, 18 or 24) refers to the sampling time (early morning, noon, early night and midnight respectively) and then to the depth stratum. The abundance of the cnidarians, ctenophores and thaliaceans is shown as the average of the three replicates and is expressed in ind. 10 m^-3^, while for the other zooplankton groups data refers to the first replicate and is expressed as ind. m^-3^ (see Material and Methods). Temperature is in °C, PAR in μE m^-2^ s^-1^ and chlorophyll-*a* in mg m^-3^.
(XLSX)

S1 Table Medusae species list with averaged water column integrated density (ind. 10 m^-3^) and averaged weighted mean depth (m) during diurnal and nocturnal hauls, frequency of capture (FC, %) and relative abundance (RA, %). SD = standard deviation, t = statistic parameter of t test; * p <0.05, **p<0.01, ***p<0.001.
(PDF)

S2 Table Siphonophores species list and summary of the catches. DS = developmental stages; P = polygastric, E = eudoxid. Other legends as in S1 Table.
(PDF)

S3 Table Ctenophores species list and summary of the catches. Legends as in S1 Table.
(PDF)

S4 Table Thaliaceans species list and summary of the catches. DS = developmental stage; B = blastozooids (aggregates); O = oozooids (solitaries), P = phorozooids; G = gonozooids; N = nurses; A = all stages combined. Other legends as in S1 Table.
(PDF)

## References

[1] Vinogradov ME. Vertical distribution of the oceanic zooplankton Jerusalem: Israel Program for Scientific Translations; 1970.

[2] Moreira GS. On the diurnal vertical migration of Hydromedusae off Santos Brazil. Publ Seto Mar Biol Lab. 1973; 20: 537–566.

[3] Andersen V, Sardou J. *Pyrosoma atlanticum* (Tunicata, Thaliacea): diel migration and vertical distribution as a function of colony size. J Plankton Res. 1994; 16(4): 337–349.

[4] Hays GC. A review of the adaptative significance and ecosystem consequence of zooplankton diel vertical migrations. Hydrobiologia. 2003; 503: 163–170.

[5] Pearre S. Eat and run? The hunger/satiation hypothesis in vertical migration: history, evidence and consequences. Biol Rev. 2003; 78: 1–79. 12620061 10.1017/s146479310200595x

[6] Cohen JH, Forward RB Jr. Zooplankton diel vertical migration—a review of proximate control. Oceanogr mar Biol A Rev. 2009; 47: 77–110.

[7] Pagès F, Gili J-M. Vertical distribution of epipelagic siphonophores at the confluence between Benguela waters and the Angola Current over 48 hours. Hydrobiologia. 1991; 216/217: 355–362.

[8] Pagès F, Gili J-M. Influence of the thermocline on the vertical migration of medusae during a 48h sampling period. S Afr J Zool. 1992; 27(2): 50–59.

[9] Frank TM, Widder EA The correlation of downwelling irradiance and staggered vertical migration patterns of zooplankton in Wilkinson Bay, Gulf of Maine. J Plankton Res. 1997; 19(12): 1975–1991.

[10] Hays GC, Kennedy H, Frost BW Individual variability in diel vertical migration of a marine copepod: Why some individuals remain at depth when others migrate. Limnol Oceanogr. 2001; 46(8): 2050–2054.

[11] Neumann-Leitão S, Sant’anna EME, Gusmão LMO, do Nascimento-Vieira DA, Paranaguá MN, Schwamborn R. Diversity and distribution of the mesozooplankton in the tropical Southwestern Atlantic. J Plankton Res. 2008; 30(7): 795–805.

[12] Boltovskoy D, Correa N, Boltovskoy A. Marine zooplanktonic diversity: a view from the South Atlantic. Oceanol Acta. 2003; 25: 271–278.

[13] Pierrot-Bults AC, van der Spoel S. Macrozooplankton diversity: how much do we really know? Zool Verh. 2003; 345: 297–312.

[14] Boero F, Bouillon J, Gravili C, Miglietta MP, Parsons T, Piraino S Gelatinous plankton: irregularities rule the world (sometimes). Mar Ecol Prog Ser. 2008; 356: 299–310.

[15] Andersen V. Salp and pyrosomid blooms and their importance in biogeochemical cycles In: Bone Q, editor. The Biology of pelagic Tunicates, New York: Oxford University Press 1998 pp. 125–137.

[16] Russell FS. The vertical distribution of marine macroplankton. An observation on diurnal changes. J Mar Biol Assoc UK. 1925; 13(4): 769–809.

[17] Hirota J. Quantitative natural history of *Pleurobrachia bachei* in La Jolla Bight. Fish Bull. 1974; 72(2): 295–335.

[18] Andersen V, Sardou J, Nival P. The diel migrations and vertical distributions of zooplankton and micronekton in the Northwestern Mediterranean Sea. 2. Siphonophores, hydromedusae and pyrosomids. J Plankton Res. 1992; 14(8): 1155–1169.

[19] Vereshchaka AL. Small-Scale vertical distribution and behavior of the ctenophore *Beroe* in the Black Sea off Gelendzhik. Oceanol. 2002; 42(6): 811–814.

[20] Lucic D, Benovic A, Morovic M, Batistic M, Onofri I. Diel vertical migration of medusae in the open Southern Adriatic Sea over a short time period (July 2003). Mar Ecol. 2009; 30: 16–32.

[21] Amaral WJA, Montú MA, Gloeden MI. Salpidae (Thalicea) da plataforma continental do extremo sul do Brasil: composição, distribuição e abundância. Atlântica. 1997; 19: 51–66.

[22] Oliveira OMP, Araújo EM, Ayon, P, Cepeda, AA, Galea H, Genzano G, et al. Census of the Medusozoa, Ceriantharia and Ctenophora from South America. Third International Jellyfish Blooms Symposium. Mar del Plata: Universidad Nacional de Mar del Plata; 2010. p.85.

[23] Brandini FP, Nogueira M Jr, Simião M, Codina JCU, Noernberg MA. Deep chlorophyll maximum and plankton community response to oceanic bottom intrusions on the continental shelf in the South Brazilian Bight. Cont Shelf Res. 2014; 89:61–75.

[24] Matsuura Y. Contribuição ao estudo da estrutura oceanográfica da região sudeste entre Cabo Frio (RJ) e Cabo de Santa Marta Grande (SC). Ciênc. Cult. 1986; 38(8): 1439–1450.

[25] Castro BM, Lorenzzetti JÁ, Silveira ICA, Miranda LB. Estrutura termohalina e circulação na região entre o Cabo de São Tomé (RJ) e o Chuí (RS) In: Rossi-Wongtschowski CLB, Madureira LSP, editors. O ambiente oceanográfico da plataforma continental e do talude na região Sudeste-Sul do Brasil. São Paulo: Editora da Universidade de São Paulo; 2006 pp. 11–120.

[26] Boltovskoy D. South Atlantic Zooplankton. Leiden: Backhuys Publishers; 1999.

[27] Guerrero E, Gili J-M, Rodriguez C, Araújo EM, Canepa A, Calbet A, Genzano G, Mianzan HW, González RA. Biodiversity and distribution patterns of planktonic cnidarians in San Matías Gulf, Patagonia, Argentina. Mar Ecol. 2013; 34(Suppl. 1): 71–82.

[28] Nagata RM, Nogueira Júnior M, Haddad MA, Brandini FP. Spatial and temporal variation of planktonic cnidarian density in subtropical waters of the Southern Brazilian Bight. J Mar Biol Assoc UK. 2014;94(7):1387–1400.

[29] Saltzman J, Wishner KF. Zooplankton ecology in the eastern tropical Pacific oxygen minimum zone above a seamount: 2. Vertical distribution of copepods. Deep-Sea Res I. 1997; 44: 931–954.

[30] Palma S, Apablaza P. Abundancia y distribución vertical del zooplancton gelatinoso carnívoro en una área de surgencia en el norte del Sistema de la Corriente de Humboldt. Invest 3 2004; 32(1): 47–70.

[31] Tew KS, Lo W-T. Distribution of Thaliacea in SW Taiwan coastal water in 1997, with special reference to *Doliolum denticulatum*, *Thalia democratica* and *T*. *orientalis* . Mar Ecol Prog Ser. 2005; 292: 181–193.

[32] Tamaki A, Mandal S, Agata Y, Aoki I, Suzuki T, Kanehara H, Aoshima T, Fukuda Y, Tsukamoto H, Yanagi T. Complex vertical migration of larvae of the ghost shrimp, *Nihonotrypaea harmandi*, in inner shelf waters of western Kyushu, Japan. Est Coast Shelf Sci. 2010; 86: 125–136.

[33] Zar JH. Biostatistical Analysis. New Jersey: Pearson Prentice Hall; 2010.

[34] Anderson MI. A new method for non-parametric multivariate analysis of variance. Austral Ecol; 26: 32–46.

[35] Anderson MJ, Gorley RN, Clarke KR. PERMANOVA+ for PRIMER: Guide to Software and Statistical Methods Plymouth: PRIMER-E; 2008.

[36] Legendre P, Legendre L. Numerical ecology 2nd ed. Amsterdam: Elsevier Science; 1998.

[37] Lepš J, Šmilauer P. Multivariate analysis of ecological data using CANOCO Cambridge: Cambridge University Press; 2003.

[38] Lopes RM. Marine zooplankton studies in Brazil—a brief evaluation and perspectives. An Acad Bras Ciênc. 2007; 79(3): 369–379. 17768529 10.1590/s0001-37652007000300002

[39] Nogueira Júnior M, Brandini FP, Codina JCU Distribution of planktonic cnidarians in response to South Atlantic Central Water intrusion in the South Brazilian Bight. Cont Shelf Res. 2014; 89: 93–102.

[40] Musayeva EI. Distribution of siphonophores in the eastern part of the Indian Ocean. Trans Inst Oceanol. 1976; 105: 171–197.

[41] Vannucci M. On the ecology of Brazilian medusae at 25°Lat. S. Bol Inst Oceanogr. 1963; 13(1): 143–184.

[42] Daniel R. Vertical distribution of siphonophora in relation to thermocline in the Arabian Sea and Southwest Indian Ocean. Special Publications UNESCO/NIO, Proc. Symp Warm Water Zooplankton. 1977 pp: 124–127.

[43] Musayeva EI. Distribution of siphonophores in the East Indian Ocean from July to November 1962. Oceanologyia. 1971; 11(6): 1098–1103.

[44] Apablaza P, Palma S. Efecto de la zona de mínimo oxígeno sobre la migración vertical de zooplancton gelatinoso en la bahía de Mejillones. Invest 3 2006; 34(2): 83–95.

[45] Palma S. Migración nictemeral del macroplancton gelatinoso de la bahía de Villefranche-sur-Mer, Meditteráneo Noroccidental. Invest Pesq. 1985; 49(2): 261–274.

[46] Bigelow HB, Sears M. Siphonophorae. Report on the Danish Oceanographical Expeditions 1908–10 to the Mediterranean and adjacent Seas. 1937; 11 (Biology): 1–144.

[47] Patriti G. Les Siphonophores Calycophores du Golfe de Marseille. Rec Trav Stat Mar Endoume. 1964; 35(51): 185–258.

[48] Lucic D, Benovic A, Batistic M, Morovic M, Onofri I, Molinero JC, Kogovsek T, Gangai B, Miloslavic M. A short-term investigation of diel vertical migrations of the calycophoran Siphonophora in the open south Adriatic Sea (July 2003). Acta Adriat. 2011; 52(2): 159–172.

[49] Moore HB. Plankton of the Florida Current II. Siphonophora. Bull Mar Sci Gulf Caribb. 1953; 2(4): 559–573.

[50] Pugh PR. The vertical distribution of the siphonophores collected during the SOND Cruise, 1965. J Mar Biol Assoc UK. 1974; 54: 25–90.

[51] Sardou J, Etienne M, Andersen V. Seasonal abundance and vertical distributions of macroplankton and micronekton in the Northwestern Mediterranean Sea. Oceanol Acta. 1996; 19(6): 645–656.

[52] Andersen V, François F, Sardou J, Picheral M, Scotto M, Nival P. Vertical distributions of macroplankton and micronekton in the Ligurian and Tyrrhenian Seas (northwestern Mediterranean). Oceanol Acta. 1998; 21(5):655–676.

[53] Vinogradov ME, Flint MV, Shushkina EA. Vertical distribution of mesoplankton in the open area of the Black Sea. Mar Biol. 1985; 89: 95–107.

[54] Mutlu E. Distribution and abundance of ctenophores, and their zooplankton food in the Black Sea. II. *Mnemiopsis leidyi* . Mar Biol. 1999; 135:603–613.

[55] Mutlu E, Bingel F. Distribution of ctenophores, and their zooplankton food in the Black Sea. I. *Pleurobrachia pileus* . Mar Biol. 1999; 135: 589–601.

[56] Purcell JE, Shiganova T, Decker MB, Houde ED. The ctenophore *Mnemiopsis* in native and exotic habitats: U.S. Estuaries *versus* the Black Sea basin. Hydrobiologia. 2001; 451: 145–176.

[57] Roe HSJ, James PT, Thurston MH. The diel migrations and distribution within a mesopelagic community in the North East Atlantic. 6. Medusae, Ctenophores, Amphipods and Euphasiids. Prog Oceanogr. 1984; 13: 425–460.

[58] Haraldsson M, Bamstedt U, Tiselius P, Titelman J, Aksnes DL. Evidence of diel vertical migration in *Mnemiopsis leidyi* . PLOS One. 2014; 9(1): e86595 doi: 10.1371/journal.pone.0086595 24466162 10.1371/journal.pone.0086595 PMC3899294

[59] Esnal GB, Daponte MC. Doliolida In: Boltovskoy D, editor. South Atlantic Zooplankton. Leiden: Backhuys Publishers; 1999 pp. 1409–1421.

[60] Paffenhöfer G-A, Atkinson LP, Lee TN, Verity PG, Bulluck LR. Distribution and abundance of thaliacenas and copepods off the southeastern U.S.A. during winter. Cont Shelf Res. 1995; 15(2/3): 255–280.

[61] Ménard F, Fromentin J-M, Goy J, Dallot S. Temporal fluctuations of doliolid abundance in the bay of Villefranche-sur-Mer (Northwestern Mediterranean Sea) from 1967 to 1990. Oceanol Acta. 1997; 20(5): 733–742.

[62] Berner LD, Reid JL. On the response to changing temperature of the temperature-limited plankter *Doliolum denticulate* Quoy and Gaimard 1835. Limnol Oceanogr. 1961; 6(2): 205–215.

[63] Tsuda A, Nemoto T. Distribution and growth of salps in a Kuroshio warm-core ring during summer 1987. Deep-Sea Res. 1992; 39(1): 219–229.

[64] Gibbons MJ. Vertical distribution and feeding of *Thalia democratica* on the Agulhas Bank during march 1994. J Mar Biol Assoc UK. 1997; 77: 493–505.

[65] Andersen V, Gubanova A, Nival P, Ruellet T. Zooplankton community during the transition from spring bloom to oligotrophy in the open NW Mediterranean and effects of wind events. 2. Vertical distributions and migrations. J Plankton Res. 2001; 23(3): 243–261.

[66] Wiebe PH, Madin LP, Haury LR, Harbison GR, Philbin LM. Diel vertical migration by *Salpa aspera* and its potential for large-scale particulate organic matter transpoerted to the deep-sea. Mar Biol. 1979; 53: 249–255.

[67] Harbison GR, Campenot RB. Effects of temperature on the swimming of salps (Tunicata, Thaliacea): implications for vertical migrations. Limnol Oceanogr. 1979; 24(6): 1081–1091.

[68] Chae J, Choi HW, Lee WJ, Kim D, Lee JH. Distribution of a pelagic tunicate, *Salpa fusiformis* in warm surface current of the eastern Korean waters and its impingement on cooling water intakes of Uljin nuclear power plant. J Environ Biol. 2008; 29(4): 585–590. 19195402

[69] Laval P, Braconnot JC, da Silva NL. Deep planktonic filter-feeders found in the aphotic zone with the Cyana submersible in the Ligurian Sea (NW Mediterranean). Mar Ecol Prog Ser. 1992; 79: 253–241.

[70] Mills CE. Vertical migration and diel activity patterns of hydromedusae: studies in a large tank. J Plankton Res. 1983; 5(5): 619–635.

[71] Arkett A. The shadow response of a hydromedusan (*Polyorchis penicillatus*): behavioral mechanisms controlling diel and ontogenic vertical migration. Biol Bull. 1985; 169: 297–312. 29314927 10.2307/1541483

[72] Larson RJ. Obervations on the light-inhibited activity cycle and feeding behavior of the hydromedusa *Olindias tenuis* . Stud Fauna Curaçao Caribbean Isl. 1986; 213: 191–199.

[73] Falkenhaug T. Distributional and seasonal patterns of ctenophores in Malagen, northern Norway. Mar Ecol Prog Ser. 1996; 140: 59–70.

[74] Anderson PAV. The comparative electrobiology of gelatinous zooplankton. Bull Mar Sci. 1985; 37(2): 460–477.

[75] Anderson PAV, Mackie GO. Electrically coupled photosensitive neurons control swimming in a jellyfish. Science. 1977; 197: 186–188. 17918 10.1126/science.17918

[76] Martin VJ. Photoreceptors of cnidarians. Can J Zool. 2002; 80: 1703–1722.

[77] Sainmont J, Thygesen UH, Visser AW. Diel vertical migration arising in a habitat selection game. Theor Ecol., 2013; 6:241–251.

[78] Pearre S. Problems of detection and interpretation of vertical migration. J Plankton Res., 1979; 1(1):29–44.

[79] Ralston DK, Mcgillicuddy DJ, Townsend DW. Asynchronous vertical migration and bimodal distribution of motile phytoplankton. J Plankton Res., 2007; 29(9):803–821.

[80] Hardy AC, Paton WN. Experiments on the vertical migration of plankton animals. J Mar Biol Assoc UK., 1947; 26:467–526. 10.1017/s0025315400013722 20249678

[81] Cottier FR, Tarling GA, Wold A, Falk-Petersen S. Unsynchronized and synchronized vertical migration of zooplankton in a high arctic fjord. Limnol Oceanogr., 2006; 51(6):2586–2599.

[82] Baumgartner MF, Lysiak NSJ, Schuman C, Urban-Rich J., Wenzel FW. Diel vertical migration behavior of *Calanus finmarchicus* and its influence on right and sei whale occurrence. Mar Ecol Progr Ser., 2011; 423: 167–184.

[83] Kaartvedt S, Titelman J, Røstad A, Klevjera TA. Beyond the average: Diverse individual migration patterns in a population of mesopelagic jellyfish. Limnol Oceanogr., 2011; 56(6): 2189–2199.

[84] Liu S-H, Sun S, Han B-P. Diel vertical migration of zooplankton following optimal food intake under predation. J Plankton Res., 2003; 25(9):1069–1077.

[85] Sekino T, Yamamura N. Diel vertical migration of zooplankton: optimum migrating schedule based on energy accumulation. Evol Ecol., 1999; 13:267–282.

[86] Huntley M, Brooks ER. Effects of age and food availability on diel vertical migration of *Calanus pacificus* . Mar Biol., 1982; 71:23–31.

[87] Alves Junior N, Meirelles PM, Santos EO, Dutilh B, Silva GGZ, Paranhos R, et al Microbial community diversity and physical–chemical features of the Southwestern Atlantic Ocean. Arch Microbiol., 2014; doi: 10.1007/s00203-014-1035-6 10.1007/s00203-014-1035-6 25205422

[88] Rinke K, Hübner I, Petzoldt T, Rolinski S, König-Rinke M, Post J, et al How internal waves influence the vertical distribution of zooplankton. Freshwat Biol. 2007; 52:137–144.

[89] Huber AMR, Peeters F, Lorke A. Active and passive vertical motion of zooplankton in a lake. Limnol Oceanogr. 2011; 56(2): 695–706.

[90] Pernica P, Wells MG, Sprules WG. Internal waves and mixing in the epilimnion of a lake affects spatial patterns of zooplankton in a body-size dependent manner. Limnol Oceanogr, Fluids Environ. 2013; 3: 279–294.

[91] Macias D, Somavilla R, González-Gordillo JI, Echevarria F. Physical control on zooplankton distribution pattern at the Strait of Gibraltar during an episode of internal wave generation. Mar Ecol Prog Ser. 2010; 408: 79–95.

[92] Johannenssen OM. Note on some hydrographical and current observations from three positions on the Brazilian shelf in the region of Cabo Frio-Santos 1966. Contrib Avulsas Inst Oceanogr. 1968; 10:1–8.

[93] Uye S, Huang C, Onbe T. Ontogenetic diel vertical migration of the planktonic copepod *Calanus sinicus* in the Inland Sea of Japan. Mar Biol. 1990; 104:389–396.

[94] Pugh PR. The diel migrations and distribution within a mesopelagic community in the North East Atlantic. 7. Siphonophores. Prog Oceanogr. 1984; 13:461–489.

[95] Pagès F, Kurbjeweit F. Vertical distribution and abundance of mesoplanktonic medusae and siphonophores from the Weddell Sea, Antarctica. Polar Biol. 1994; 14: 243–251.

[96] Gibson DM, Paffenhöfer G-A. Feeding and growth rates of the doliolid, *Dolioletta gegenbauri* Uljanin (Tunicata, Thaliacea). J Plankton Res. 2000; 22(8): 1485–1500.

[97] Gibson DM, Paffenhöfer G-A. Asexual reproduction of the doliolid *Dolioletta gegenbauri* Uljanin (Tunicata, Thaliacea). J Plankton Res. 2002; 24(7): 703–712.

[98] Schlitzer R. Ocean Data View. 2012. Available: http://www.odv.awi.de. Accessed 17 October 2015.

