# Supplementary material for: Diel Vertical Dynamics of Gelatinous Zooplankton (Cnidaria, Ctenophora and Thaliacea) in a Subtropical Stratified Ecosystem (South Brazilian Bight)
Source: PLoS One. 2015 Dec 4;10(12):e0144161. doi: 10.1371/journal.pone.0144161 (PMC4670095; doi:10.1371/journal.pone.0144161)
Supplement: S1 Table — SD = standard deviation, t = statistic parameter of t test; * p <0.05, **p<0.01, ***p<0.001. (PDF) [file pone.0144161.s002.pdf]

Diel vertical dynamics of gelatinous zooplankton (Cnidaria, Ctenophora and Thaliacea) in a subtropical stratified ecosystem (South Brazilian Bight)

Miodeli Nogueira Júnior*, Frederico P Brandini & Juan Carlos Ugaz Codina

*miodeli@gmail.com

S1 Table. Medusae species list with averaged water column integrated density (ind.*10 m^{-3}) and averaged weighted mean depth (m) during diurnal and nocturnal hauls, frequency of capture (FC, %) and relative abundance (RA, %). SD = standard deviation, t = statistic parameter of t test; * p <0.05, **p<0.01, ***p<0.001.

Taxa	Average density (±SD)		FC	RA	Weighted mean depth (±SD)		
	Day	Night			Day	Night	t
**HYDROZOA**							
Actinula larvae	0.09 (±0.1)	0.04 (±0.09)	4.2	0.3	52.5 (±46)	85	-
**Anthoathecata**							
Bougainvillia frondosa Mayer, 1900	0.09 (±0.17)	0.04 (±0.09)	4.2	0.2	20	55	-
Corymorpha gracilis (Brooks, 1882)	1.8 (±1.3)	2.6 (±2.4)	41.7	7.8	51.7 (±5.5)	31.1 (± 16.1)	2.41
Ectopleura dumortierii (van Beneden, 1844)	0	0.04 (±0.09)	1.4	0.1	-	55	-
Zanclea sp.	0.09 (±0.1)	0	2.8	0.2	79 (±21.2)	-	-
**Lepthothecata**							
Obelia spp.	0	0.04 (±0.09)	1.4	0.1	-	20	-
Mitrocomium cirratum Haeckel, 1879	0.04 (±0.09)	0	1.4	0.1	85	-	-
**Limnomedusae**							
Proboscidactyla ornata (McCrady, 1859)	0.43 (±0.54)	0.13 (±0.26)	16.7	1.5	31.7 (±20.2)	34	-
**Narcomedusae**							
Bitentaculata larvae	0.39 (±0.5)	0.13 (±0.09)	12.5	1.0	30 (±14.1)	41.7 (±37.5)	0.4
Cunina octonaria McCrady, 1859	0.22 (±0.26)	0.13 (±0.17)	8.3	0.6	37 (±4.3)	20	-
Solmaris corona (Keferstein & Ehlers, 1861)	0.3 (±0.2)	1.57 (±1.1)	25.0	3.2	51.4 (±28.5)	20	0.07
Aegina citrea Eschscholtz, 1829	0.09 (±0.1)	0.04 (±0.08)	2.8	0.1	-	20	-
Solmundella bitentaculata (Quoy & Gaimard, 1833)	0.35 (±0.25)	0.7 (±0.62)	22.2	2.1	73.9 (±14.3)	38.3 (±31.4)	2.07
**Trachymedusae**							
Aglaura hemistoma Péron & Lesueur, 1810	24.1 (±27)	8.8 (±3.5)	79.2	69.1	57.9 (±9.1)	27.2 (±4)	6.2***
Liriope tetraphylla (Chamisso & Eysenhardt, 1821)	1.9 (±2)	2.2 (±1.3)	52.8	8.0	54 (±8.2)	31.7 (±5.9)	4.38**
Rhopalonema velatum Gegenbaur, 1857	0.3 (±0.39)	0.09 (±0.1)	11.1	0.84	69.2 (±15.1)	20	-
Rhopalonematidae sp.1 (*Arctapodema*?)	1.6 (±1.4)	0.4 (±0.46)	20.8	3.5	25.6 (± 4.9)	20	0.07
**SCYPHOZOA**							
**Coronatae**							
Nausithoe aurea Silveira & Morandini, 1997	0.22 (±0.26)	0.13 (±0.17)	9.7	0.7	65.2 (±27.9)	20	-
Nausithoe punctata Kölliker, 1853	0.09 (±0.1)	0.04 (±0.09)	4.2	0.3	70.5 (±21.9)	20	-
