# Supplementary material for: Diel Vertical Dynamics of Gelatinous Zooplankton (Cnidaria, Ctenophora and Thaliacea) in a Subtropical Stratified Ecosystem (South Brazilian Bight)
Source: PLoS One. 2015 Dec 4;10(12):e0144161. doi: 10.1371/journal.pone.0144161 (PMC4670095; doi:10.1371/journal.pone.0144161)
Supplement: S2 Table — DS = developmental stages; P = polygastric, E = eudoxid. Other legends as in S1 Table. (PDF) [file pone.0144161.s003.pdf]

Diel vertical dynamics of gelatinous zooplankton (Cnidaria, Ctenophora and Thaliacea) in a subtropical stratified ecosystem (South Brazilian Bight)

Miodeli Nogueira Júnior*, Frederico P Brandini & Juan Carlos Ugaz Codina

*miodeli@gmail.com

S2 Table. Siphonophores species list and summary of the catches. DS = developmental stages; P = polygastric, E = eudoxid. Other legends as in S1 Table.

Taxa	DS	Average density (±SD)		FC	RA	Weighted mean depth (±SD)		
		Day	Night			Day	Night	t
**SIPHONOPHORAE**								
Destroyed, unidentified		0.4 (±0.6)	0.4 (±0.6)	**16.7**	**1.5**			
**Physonectae**								
Athorybia larvae		0.17 (±0.14)	0.26 (±0.22)	**13.9**	**0.86**	48.3 (±33.3)	19.9 (±17.6)	0.55
Agalma elegans (Sars, 1846)		0.04 (±0.09)	0	**1.4**	**0.08**	20	-	-
Agalma okeni Eschscoltzi, 1829		0	0.04 (±0.09)	**1.4**	**0.08**	-	55	-
Cordagalma ordinatum (Haeckel, 1888)		0.35 (±0.3)	0.5 (±0.36)	**25**	**1.6**	66.7 (±15.8)	42.2 (±15.4)	2.06
Nanomia bijuga (Delle Chiaje, 1841)		0.35 (±0.3)	0.4 (±0.3)	**22.2**	**1.6**	61.9 (±7.6)	53.8 (±29.2)	0.47
Physophora hydrostatica Forskål, 1775		0.09 (±0.1)	0.13 (±0.09)	**6.9**	**0.4**	55	75 (±17.3)	0.21
**Calycophorae**								
Amphicaryon acaule Chun, 1888	**P**	0.04 (±0.09)	0	**1.4**	**0.08**	85	-	-
Chelophyes appendiculata (Eschscholtz, 1829)	**P**	0.17 (±0.25)	0.26 (±0.3)	**13.9**	**0.86**	42.8 (±32.2)	43.9 (±20.7)	0.05
	**E**	0.7 (±0.26)	0.4 (±0.46)	**22.2**	**2.32**	26.8 (±9.4)	22.5 (±4.2)	0.73
Diphyes bojani (Eschscholtz, 1825)	**P**	2.9 (±1.7)	1.6 (±0.5)	**54.2**	**8.9**	43.4 (±7.3)	24.3 (±8.7)	3.3*
	**E**	6.8 (±4.4)	3.7 (±1.4)	**52.8**	**20.8**	39.8 (±7.1)	21.3 (±1.7)	5.1**
Diphyes dispar Chamisso & Eysenhardt, 1821	**P**	0.04 (±0.09)	0.04 (±0.09)	**2.8**	**0.17**	20	20	-
	**E**	0.09 (±0.1)	0.26 (±0.2)	**9.7**	**0.69**	37.5 (±24.7)	32.8 (±22.2)	0.22
	**A**	0.13 (±0.17)	0.3 (±0.26)	**9.7**	**0.86**	37.5 (±24.7)	29.1 (±15.8)	0.45
Eudoxoides mitra (Huxley, 1859)	**P**	0	0.04 (±0.09)	**1.4**	**0.08**	-	20	-
	**E**	0.2 (±0.2)	0	**6.9**	**0.43**	35.2 (±26.3)	-	-
Eudoxoides spiralis (Bigelow, 1911)	**P**	0	0.1 (±0.26)	**2.8**	**0.26**	-	20	-
	**E**	0.6 (±0.36)	0.4 (±0.8)	**12.7**	**1.89**	27 (±8.1)	20	-
Lensia subtilis (Chun, 1886)	**P**	0.09 (±0.1)	0.2 (±0.17)	**8.3**	**0.6**	52.5 (±45.9)	32.8 (±22.2)	0.67
	**E**	0.5 (±0.5)	0.1 (±0.17)	**18**	**1.29**	42.71 (±26.2)	20	0.31
Lensia sp.	**E**	0.17 (±0.14)	0.04 (±0.09)	**6.9**	**0.43**	31.7 (±20)	20	-
Muggiaea kochii (Will, 1844)	**P**	0.5 (±0.4)	0.7 (±0.4)	**20.8**	**2.41**	39.1 (±11.8)	21.8 (±3.7)	2.79*
	**E**	0.2 (±0.2)	0.26 (±0.2)	**11.1**	**0.95**	50.2 (±6.7)	20	8.34**
Sphaeronectes koellikeri Huxley, 1859	**E**	0.09 (±0.1)	0.04 (±0.09)	**4.2**	**0.26**	52.5 (±46)	85	-

S2 Table. Continued

Taxa	DS	Mean density (±SD)		FC	RA	Weighted mean depth (±SD)		
		Day	Night			Day	Night	t
Abylopsis tetragona (Otto, 1823)	**P**	2 (±1.5)	3.3 (±0.5)	**56.9**	**19.67**	39 (±7.1)	27.2 (±4.2)	2.86*
	**E**	3.6 (±0.9)	7.9 (±1.5)	**72.2**	**22.8**	64 (±5.7)	28.6 (±2.9)	11***
Abylopsis eschscholtzii (Huxley, 1859)	**P**	0.7 (±0.3)	0.6 (±0.3)	**26.4**	**2.58**	36.7 (±7.2)	21.8 (±3.7)	3.66*
	**E**	0.7 (±0.4)	1.3 (±0.2)	**26.4**	**3.96**	25 (±10)	22.9 (±5.8)	0.36
Bassia bassensis (Quoy & Gaimard, 1833)	**P**	0.09 (±0.1)	0.1 (±0.08)	**6.9**	**0.43**	37.5 (±24.8)	20	1.34
	**E**	0.35 (±0.2)	0.26 (±0.2)	**15.3**	**1.2**	33.7 (±17)	20	1.37
Enneagonum hyalinum Quoy & Gaimard, 1827	**P**	1.3 (±0.6)	1.3 (±1)	**37.5**	**5.33**	31.9 (±8.9)	26.2 (±7.3)	0.98
	**E**	0.5 (±0.4)	1.1 (±0.1)	**30.5**	**3.35**	31.5 (±10)	29.6 (±12.5)	0.22
