# Supplementary material for: Diel Vertical Dynamics of Gelatinous Zooplankton (Cnidaria, Ctenophora and Thaliacea) in a Subtropical Stratified Ecosystem (South Brazilian Bight)
Source: PLoS One. 2015 Dec 4;10(12):e0144161. doi: 10.1371/journal.pone.0144161 (PMC4670095; doi:10.1371/journal.pone.0144161)
Supplement: S3 Table — Legends as in S1 Table. (PDF) [file pone.0144161.s004.pdf]

Diel vertical dynamics of gelatinous zooplankton (Cnidaria, Ctenophora and Thaliacea) in a subtropical stratified ecosystem (South Brazilian Bight)

Miodeli Nogueira Júnior*, Frederico P Brandini & Juan Carlos Ugaz Codina

*miodeli@gmail.com

S3 Table. Ctenophores species list and summary of the catches. Legends as in S1 Table.

Taxa	Average density (±SD)		FC	RA	Weighted mean depth (±SD)		
	Day	Night			Day	Night	t
**CTENOPHORA**							
**Beroida**							
Beroe sp.	0.6 (±0.3)	1.5 (±0.8)	29.17	92.1	70.45 (±12.9)	35.5 (±23.7)	1.76*
**Lobatae**							
Destroyed unidentified	0.04 (±0.09)	-	1.39	1.97	55	-	
**Cestida**							
Velamen parallelum (Fol. 1869)	0	0.13 (±0.17)	4.17	5.88	-	20	
