# Supplementary material for: Diel Vertical Dynamics of Gelatinous Zooplankton (Cnidaria, Ctenophora and Thaliacea) in a Subtropical Stratified Ecosystem (South Brazilian Bight)
Source: PLoS One. 2015 Dec 4;10(12):e0144161. doi: 10.1371/journal.pone.0144161 (PMC4670095; doi:10.1371/journal.pone.0144161)
Supplement: S4 Table — DS = developmental stage; B = blastozooids (aggregates); O = oozooids (solitaries), P = phorozooids; G = gonozooids; N = nurses; A = all stages combined. Other legends as in S1 Table. (PDF) [file pone.0144161.s005.pdf]

Diel vertical dynamics of gelatinous zooplankton (Cnidaria, Ctenophora and Thaliacea) in a subtropical stratified ecosystem (South Brazilian Bight)

Miodeli Nogueira Júnior*, Frederico P Brandini & Juan Carlos Ugaz Codina

*miodeli@gmail.com

S4 Table. Thaliaceans species list and summary of the catches. DS = developmental stage; B = blastozooids (aggregates); O = oozooids (solitary), P = phorozooids; G = gonozooids; N = nurses; A = all stages combined. Other legends as in S1 Table.

Taxa	DS	Average density (±SD)		FC	RA	Weighted mean depth (±SD)		
		Day	Night			Day	Night	t
**THALIACEA**								
**Salpida**								
Cyclosalpa spp. (*C. bakeri* and *C. polae*?)	**B**	0.26 (±0.33)	0.09 (±0.1)	8.33	0.33	77.8 (±10.1)	20	
	**O**	0	0.04 (±0.09)	1.39	0.04	-	20	
(?) *Ritteriella amboinensis* (Apstein, 1904)	**O**	0	0.04 (±0.09)	1.39	0.04	-	55	
Salpa fusiformis Cuvier, 1804	**B**	3.44 (±3.6)	2.13 (±1.8)	41.67	5.38	56.8 (±16.8)	27.5 (±9.8)	2.93*
	**O**	1.48 (±1.8)	0.74 (±0.41)	34.72	2.13	51.7 (±21.7)	44.3 (±13.3)	0.58
Thalia democratica (Forskål, 1775)	**B**	15.15 (±17.9)	7 (±2.9)	66.67	21.23	50.9 (±10.4)	22.2 (±3.8)	5.17**
	**O**	1.48 (±1.6)	0.17 (±0.14)	26.39	1.58	54.06 (±17.7)	41.7 (±37.5)	0.59
**Doliolida**								
Dolioletta gegenbauri (Uljanin, 1884)	**P**	7.53 (±9.7)	2.6 (±1.28)	61.11	9.92	44.7 (±17.1)	36.1 (±0.9)	1
	**G**	1.22 (±1)	0.48 (±0.26)	33.33	1.67	44.5 (±18.7)	22.7 (±5.4)	2.2
	**N**	2.52 (1.18)	2.48 (±1.3)	64.28	4.9	43.5 (±5.9)	30.7 (±2.3)	4**
Doliolum nationalis Borgert, 1893	**P**	10.14 (±10.8)	3.22 (±3.05)	56.94	12.8	41 (±12.4)	32 (±5.2)	1.34
	**G**	23.46 (±24.1)	12 (±9.8)	61.11	33.99	29.9 (±3.6)	21.9 (±0.6)	4.38**
	**N**	3.39 (±2.06)	1.87 (±1.13)	55.55	5.04	45.1 (±9.9)	23.4 (±4.3)	4.03**
Destroyed, unidentified				5.55	0.54			
**Pirossomatida**								
(?) *Pyrossomella verticillata* (Neumann, 1909)		0.09 (±0.1)	0.09 (±0.1)	5.55	0.17	67.5 (±17.7)	20	
